# Archaeobotanical and isotopic analyses of waterlogged remains from the Neolithic pile-dwelling site of Zug-Riedmatt (Switzerland): Resilience strategies of a plant economy in a changing local environment

**DOI:** 10.1371/journal.pone.0274361

**Published:** 2022-09-28

**Authors:** Bigna L. Steiner, Héctor Martínez-Grau, Stefano M. Bernasconi, Eda Gross, Irka Hajdas, Stefanie Jacomet, Madalina Jaggi, Gishan F. Schaeren, Ferran Antolín

**Affiliations:** 1 Department of Environmental Sciences, Integrative Prehistory and Archaeological Science (IPAS), University of Basel, Basel, Switzerland; 2 Institute of Geology, ETH Zurich, Zürich, Switzerland; 3 Laboratory of Ion Beam Physics, ETH Zurich, Zürich, Switzerland; 4 Department of Archaeology of Canton Zug, Zug, Switzerland; 5 German Archaeological Institute, Berlin, Germany; New York State Museum, UNITED STATES

## Abstract

The excellent preservation of the waterlogged botanical remains of the multiphase Neolithic pile-dwelling site of Zug-Riedmatt (Central Switzerland) yielded an ideal dataset to delve into the issue of plant economy of a community spanning several decades. The study identified a major change in crops where oil plants played a key role in the site’s initial phase before being supplanted over the course of a few decades by naked wheat, barley and pea. Wild plants continued to be gathered albeit in different proportions. In the latest settlement phase, the changes in the local vegetation and in the values of the analyses of carbon stable isotopes suggest a less humid environment. The hypothesis is that the changes perceived in the plant economy represent a resilience strategy adopted by the inhabitants in reaction to short term local climatic alterations. The two types of soil sampling techniques (monolith and bulk) allowed comparing these results. While the density of plant remains appears to be underestimated among the samples collected by the monolith technique, the proportions of economic taxa remain unaffected. The findings thus reveal that when the bulk samplings are distributed carefully throughout multiphase sites and avoid mixing stratigraphical units, and if the samplings are representative of all archaeological features from a whole area, then each of the two techniques offer analogous results.

## 1. Introduction and aims

The attempt to grasp the resilience strategies and agricultural decision-making processes of small-scale farmers is increasingly crucial to current society as today’s industrialised agricultural systems are being questioned on the grounds of sustainability and their role in provoking climate change. Archaeological evidence indicates that Neolithic communities practiced integrated small-scale farming and rearing livestock along with intensive fishing, hunting and plant gathering ([1–4]; see also [5, 6]). There still remains much to learn as to the details of past economic systems to serve current and future small-scale farmers. The AgriChange Project ([7]) explores this question by focusing on a series of key sites in Western Europe with a high informative potential, particularly settlements yielding well-preserved waterlogged finds. This project combining archaeobotanical data, crop pest and isotope analyses, and radiocarbon datings intends to reconstruct past agricultural systems and detect factors affecting crop choice. This study focuses on Zug-Riedmatt, a Late Neolithic pile dwelling site on the shores of Lake Zug (Central Switzerland) forming part of the group of sites classified by the UNESCO World Heritage as *Prehistoric Pile Dwellings around the Alps*. The current study thus integrates research from two Swiss National Science Foundation projects: *Formation and taphonomy of archaeological wetland deposits*: *two transdisciplinary case studies and their impact on lakeshore archaeology* (Project no. 149679) and *Small seeds for large purposes*: *an integrated approach to agricultural change and climate during the Neolithic in Western Europe* (Project no. 170515).

**Fig 1:**
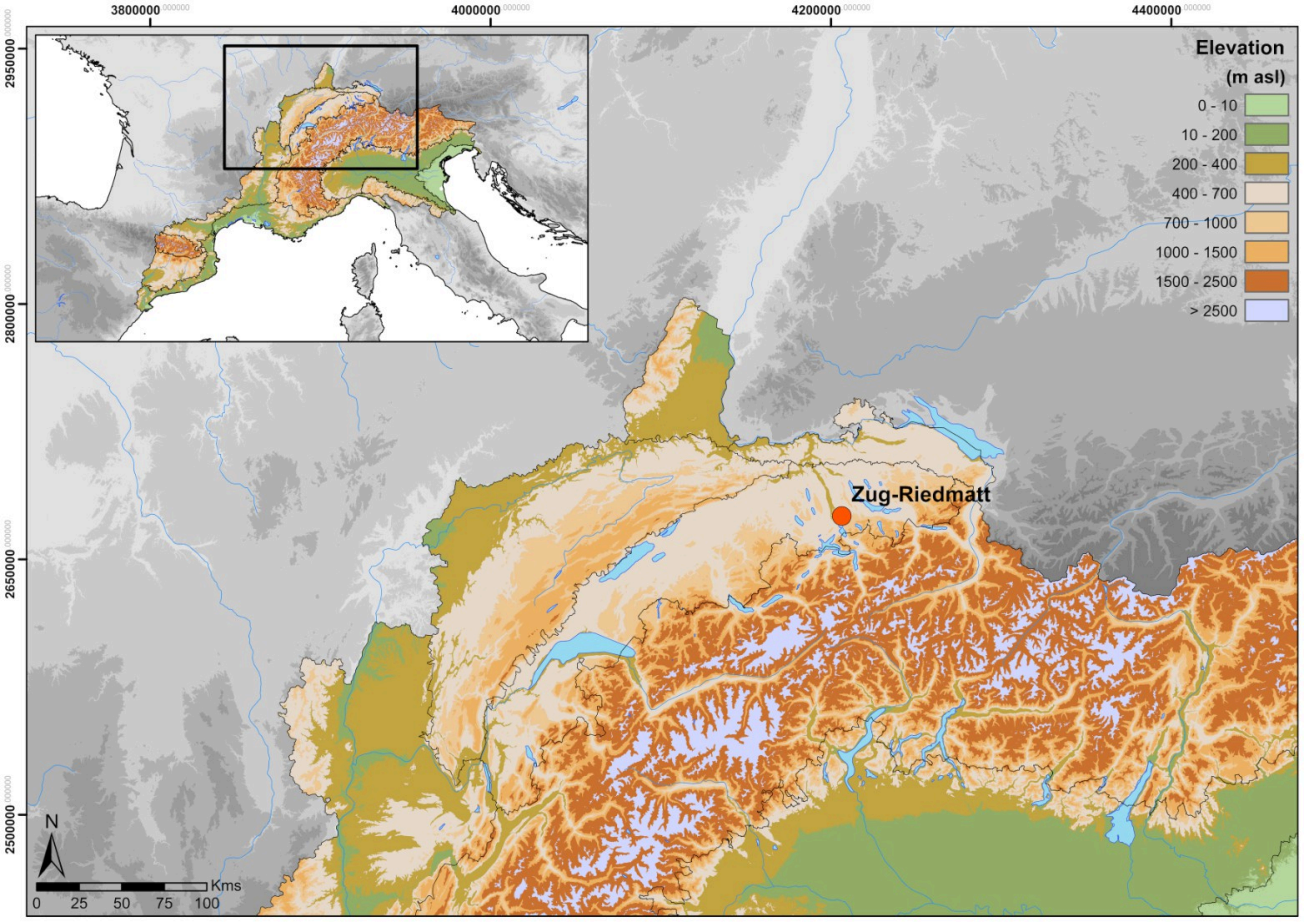
Location of the settlement of Zug-Riedmatt (Canton of Zug, Switzerland) and in colours the extension of the area of the AgriChange research project. Software: QGIS3.6, © European Union, Copernicus Land Monitoring Service [2016], European Environment Agency (EEA).

**Fig 2:**
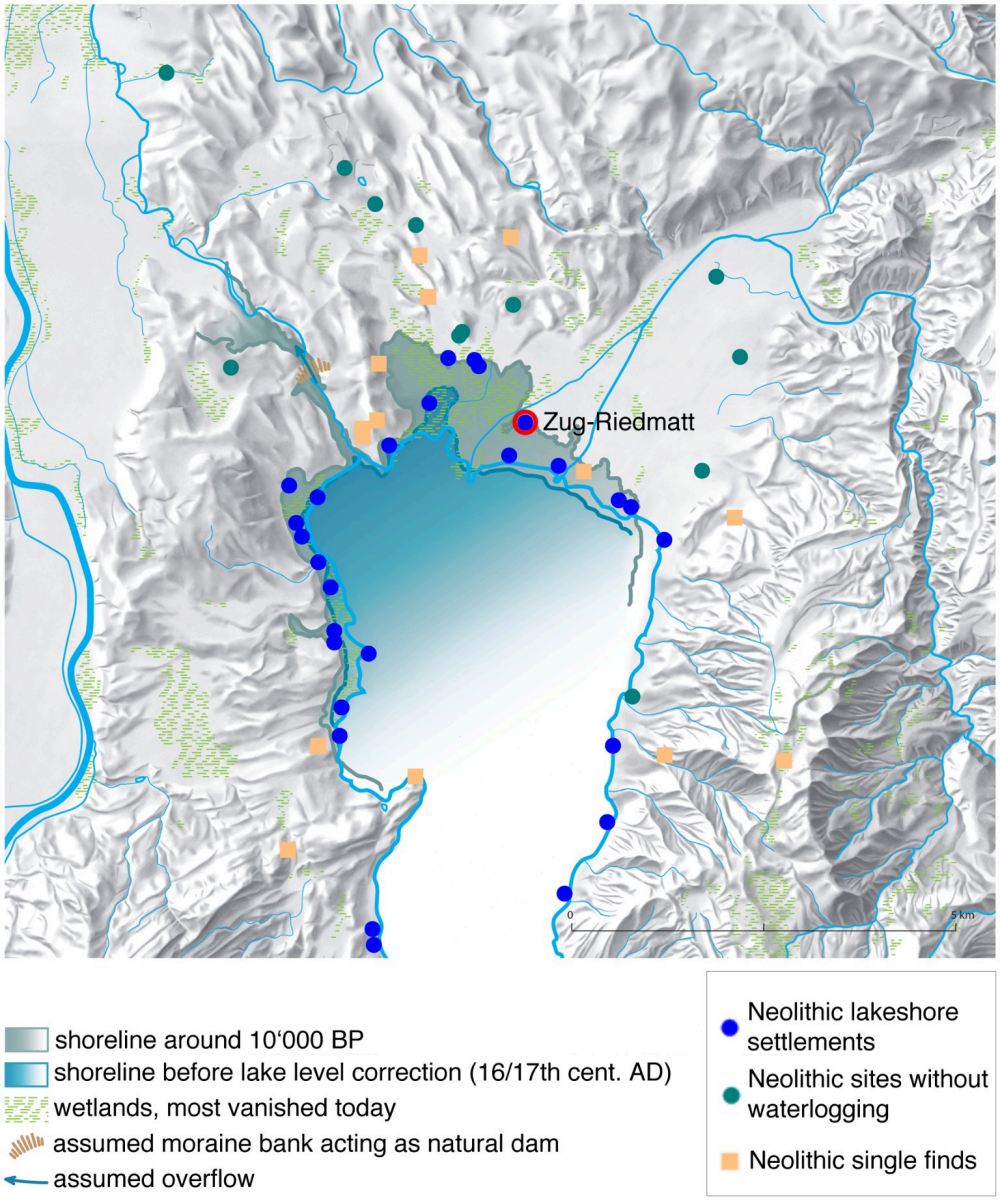
Position of Zug-Riedmatt along the shore of Lake Zug. Published under a CC BY license, with permission from Amt für Denkmalpflege und Archäologie, Zug (Eva Kläui, Salvatore Pungitore, Eda Gross, Renata Huber), original copyright 2014.

**Fig 3:**
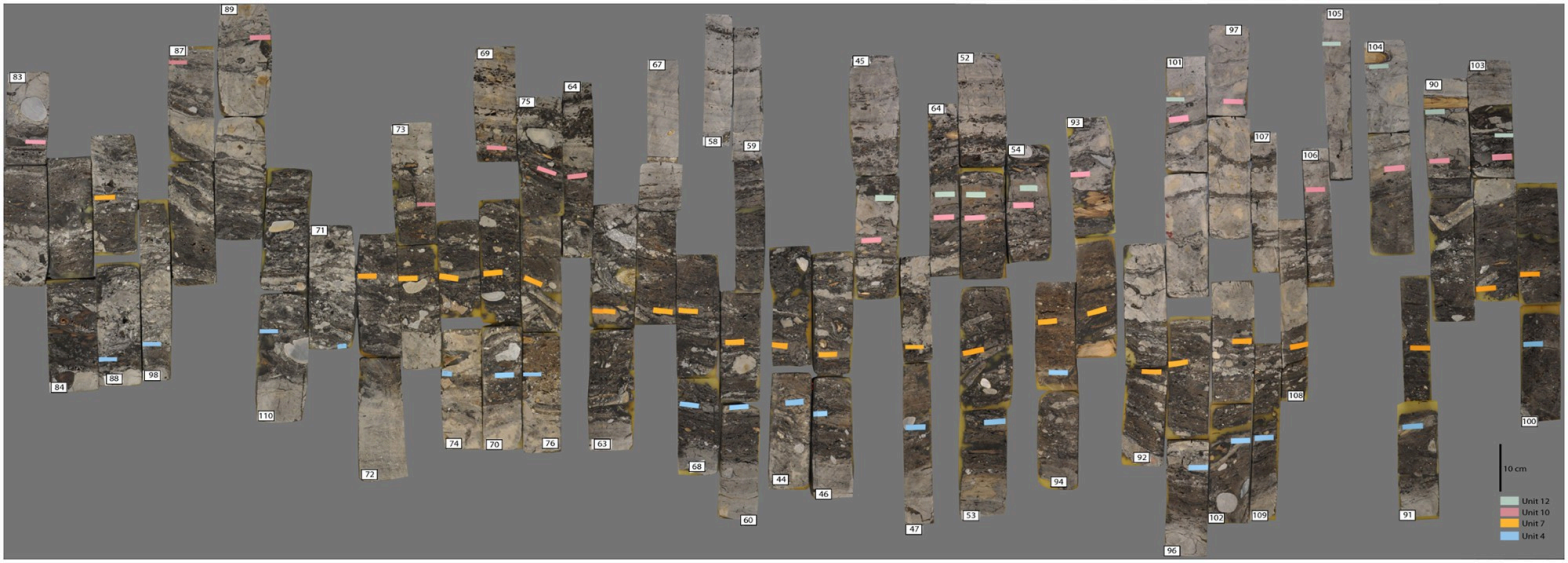
Projection onto a single plane of the different monolith column soil samples (polished sections). The organic micrite layers (units 4, 7, 10 and 12) serving as separators are indicated by different colours (©K. Ismail-Meyer). See Fig 5 for the exact position of each sample.

The excavation of ca. 64 m^2^ at Zug-Riedmatt to a depth of about 6 m brought to light several distinct layers (totalling 1.3 m in width) representing different occupations spanning a total period of ca. 150 years ([8]; Ismail-Meyer et al., in prep.; Figs 1 and 2). The excellent preservation of the organic remains stems from the anoxic waterlogged conditions. The 150 year timeframe initiated in the end of the 4th millennium cal BC was subdivided into 14 stratigraphic units (Fig 3; Table 1).

**Table 1:** Listing of the phase of occupation, settlement layers, stratigraphic units and sediment types of the site of Zug-Riedmatt. Specific stratigraphical units that are the focus of this article are marked in bold. Sediment types: orange = organic, blue = organic micrite, grey = micrite, pink = loam, white = mixed.

settlement layer	stratigraphic unit	description	hydrological characteristics
	U14	micrite above occupation phase	
	U13	‘mixed’layer	
fourth	U12	5^th^ organic micrite layer	
	U11	loam	
third	U10	4^th^ organic micrite layer	
	U9	loam	
	**U8**	**3**^**rd**^ **organic layer**	**eulittoral, landwards of reed belt. influenced by marshy waterbody**
second	U7	3^rd^ organic micrite layer	
	**U6**	**2**^**nd**^ **organic layer**	**eulittoral, reed belt, influenced by marshy waterbody/river**
first	**U5**	**loam and ‘bone midden’**	**partly sublittoral, influenced by lake/marshy waterbody**
	U4	2^nd^ organic micrite layer	
	**U3**	**1**^**st**^ **organic layer**	**partly sublittoral, influenced by lake/marshy waterbody**
	U2	1^st^ organic micrite layer	
	U1	micrite below occupation phase	

The dating of the site currently ranges from ca. 3200 to 3050 cal BC ([9]). Dendrochronology, the method most often applied to date lake-dwellings in Central Europe, [10], only served to date Zug-Riedmatt’s latest phase as wood from the earlier phases was unsuitable (for details see the materials and methods section below). The earlier phases thus had to be dated by the radiocarbon method. The calibration curve of these datings is characterised by three pronounced parallel and consecutive wiggles yielding an uncertain range ([9], 3–5). Hence the first aim of this paper was to carry out more radiocarbon datings on stratified plant macroremains and apply a Bayesian modelling to them in order to estimate more precisely the extension of the overall occupation and its different stratigraphic units. The results confirm its different levels to coincide with the Horgen Culture (ca. 3400–2800 BC; for a chronological table of the Swiss/Central European Neolithic see [11]).

This article focuses on three stratigraphic units made up mainly of organic materials (units 3, 6 and 8) and another marked by a well-preserved feature containing many bones referred to below as the ‘bone midden’ (unit 5; Table 1).

The ‘bone midden’ is exceptional as it comprises many red deer bones, fish scales and frog bones. Archaeozoological analyses of its lower features suggest it stems from an extraordinary and short-lived hunting event during the spring/early summer ([12]). Its upper section sealed by a layer of loam offers evidence, on the other hand, of recurrent fishing events throughout one or consecutive winters ([13]).

The findings of an earlier taphonomy research project (https://p3.snf.ch/project-149679) revealed that the conditions of sedimentation changed considerably throughout the site’s approximately 150 year-occupation as the lowermost layers units 3 and 5 containing the ‘bone midden’ are linked to deposits in wetter conditions than those of the second and third layers of units 6 and 8 ([8], Ismail-Meyer et al., in prep.).

The analysis of the stratigraphy of Zug-Riedmat identified three sources of water: lake, river and marsh-like waterbodies (see section 2.1 and Fig 2). The data as to the conditions in the different layers are listed in Table 1 (for details see [8]). The environmental conditions changed with time and probably influenced the taphonomy of the site to a certain degree. As only very few tests yielding experimental and modern analogue data are currently available, it is difficult to estimate the degree of influence of the different types of water sources. It is also not clear whether the changes were linked to seasonal and small-scale or large-scale climatic alterations (although the former is probably more likely; see [8]).

Towards the end of the 4^th^ millennium cal BC the plant economy of the study area comprised a combination of agriculture and plant gathering (for details see [1]). The most commonly cultivated plants in this period were cereals: emmer wheat (*Triticum dicoccon*), naked wheat (typically the tetraploid type, *Triticum durum/turgidum*), and barley (mostly of the multi-rowed naked type, *Hordeum vulgare*). Pea (*Pisum sativum*) was likewise a key legume ([3]). Flax (*Linum usitatissimum*) and large quantities of opium poppy (*Papaver somniferum*) were also significant. The most commonly gathered fruits were hazelnuts (*Corylus avellana*) and acorns (*Quercus sp*.), wild apples/pears (Maloideae) and bramble/raspberry (*Rubus fruticosus/idaeus*) ([4]). The multiple settlement layers of Zug-Riedmatt offer a general portrait of this issue and yield data on whether the ratio of cultivated and gathered plants, as well as that of different plant species, remained constant throughout the occupation or if there were shifts. The second aim of this paper is therefore to determine the ratios of crop plants and gathered fruits at Zug-Riedmatt and establish whether their use changed over time. Uncharred plant remains collected in waterlogged archaeological sites such as Zug-Riedmatt are a relatively reliable proxy for consumed plants ([14]). As there is a great variation between the numbers of seeds or fruits produced per plant by different species, it is not appropriate to directly compare their numbers. The information must therefore be garnered by the calculating the caloric input.

In order to discuss the potential climatic factors that affected cereal harvests, carbon stable isotope ratios, among other techniques, have proven to be useful indicators of water availability for crops during periods of growth ([15, 16]). Thus the third aim of this paper is to test if this is a useful parameter for the area under study and if the findings correlate with the economic dynamics observed at the site.

**Fig 4:**
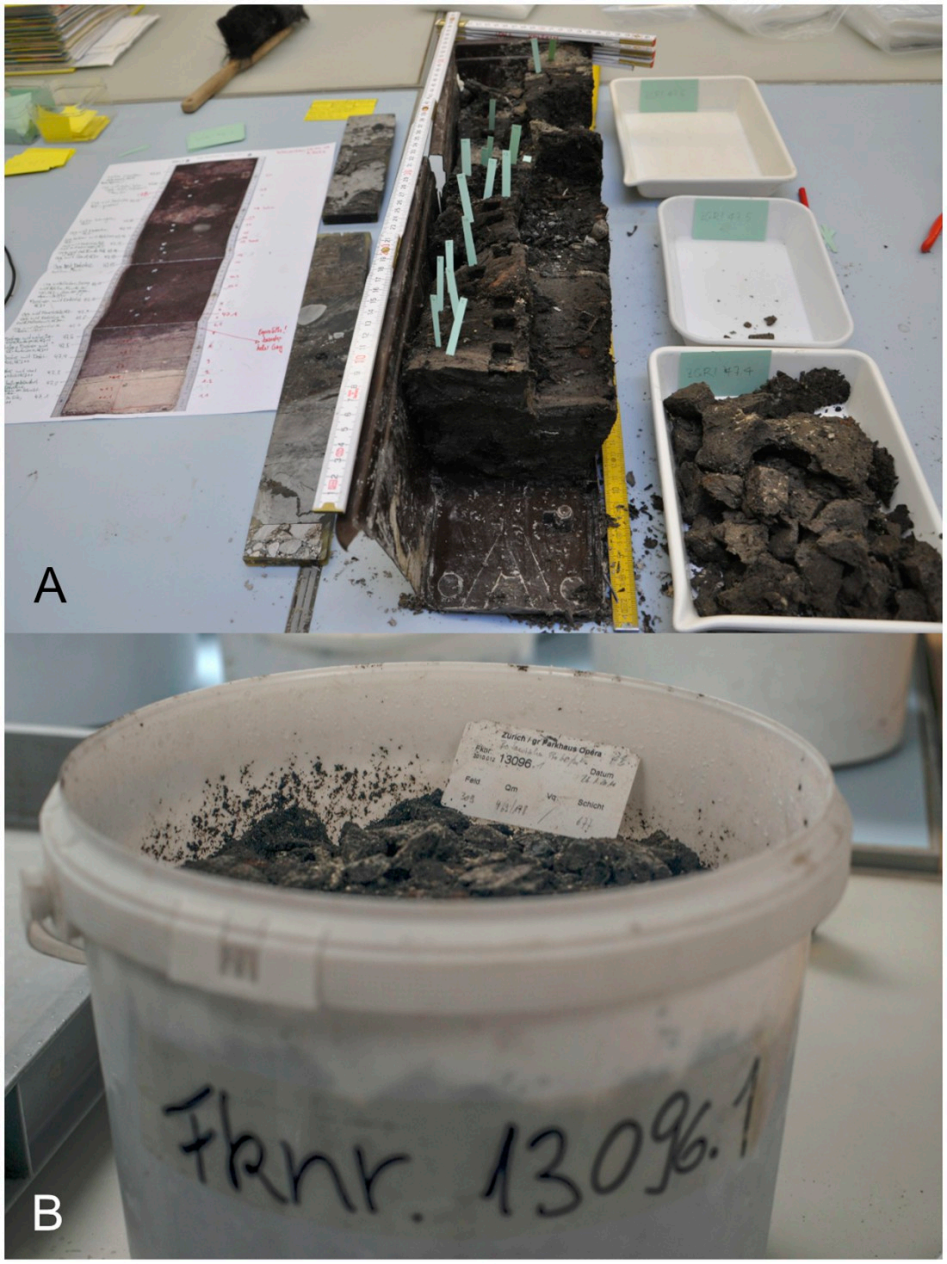
Examples of the two sampling techniques: A) small-volume monolith sample in tray (bottom right), and B) large-volume bulk sample (photo ©Raül Soteras).

The soil samples of Zug-Riedmatt, as is often the case of wetland sites, were collected by two different techniques. The small-volume monolith column were collected from the profiles of excavated sectors and subsequently separated into individual samples in the laboratory. Large-volume bulk samples, in turn, were recovered directly from loose soil removed from specific surfaces (Fig 4, see details below in the materials and methods section). Soil samples of prehistoric wetland excavations carried out in the 20^th^ century were often examined by means of the small-volume monolith method (e.g., [17–23]). The two sampling techniques today normally serve different purposes. The large-volume bulk samples are best suited to delve into palaeoeconomic issues as at least three litres of sediment is necessary to obtain representative records of all the key economic taxa ([24, 25]). Small-volume monolith samples, on the contrary, ideally serve to target layer formation processes (e.g., [8, 26]). The techniques are not mutually exclusive. Both in fact were applied during work at the Neolithic settlement of Arbon Bleiche 3 in the Canton of Thurgau ([27, 28]). Yet while the results gleaned from large and small-volume bulk samples have been compared in the past ([24, 25, 29]; see also [30, 31]), no direct systematic comparison has been undertaken of the findings stemming from large-volume bulk and small-volume monolith samples except for those of Arbon Bleiche 3 ([32], 413–414; [33], 84–85, although the sieving methods of each differed). The reason behind the lack of comparisons is that they are arduous and time-consuming to carry out especially in wetland sites with complex stratigraphic sequences.

As sampling in archaeology can greatly bias the results, it is essential to adopt the best of the different strategies ([34]). Grasping their effects and choosing the most appropriate in function of specific goals requires methodological research. A fourth aim of this study is therefore to compare the potential differences of the findings garnered from each of the two sampling techniques.

The characteristics of Zug-Riedmatt are thus ideal to delve into the dynamics of settlement layer formation as well as ancient agricultural practices and wild plant gathering behaviour over time. The site might also offer the option of determining whether a change in subsistence was influenced by natural or cultural factors by evaluating the wild plant spectrum. Furthermore, the study offers new methodological insight through its comparison of the two sampling techniques.

The aims of this paper are thus the following:

1. Narrow the chronological sequence of Zug-Riedmatt in order to gain a better grasp of the dynamics of its occupation.
2. Evaluate whether the crop types and agricultural practices at Zug-Riedmatt remained constant or not over time? And likewise, did the types of gathered fruits change over time?
3. Determine how crop management changed over time by means of carbon isotope analyses.
4. Verify whether potential changes in crop plants were provoked by taphonomic factors or by true changes of agricultural practices.
5. Assess if it is possible to compare the average density values, proportions and caloric input of cultivated and gathered fruits per layer from small-volume monolith and large-volume bulk samples.
6. Identify which plant remains are most affected if there are differences between the two sample types.

## 2. Materials and methods

### 2.1 The site of Zug-Riedmatt

**Fig 5:**
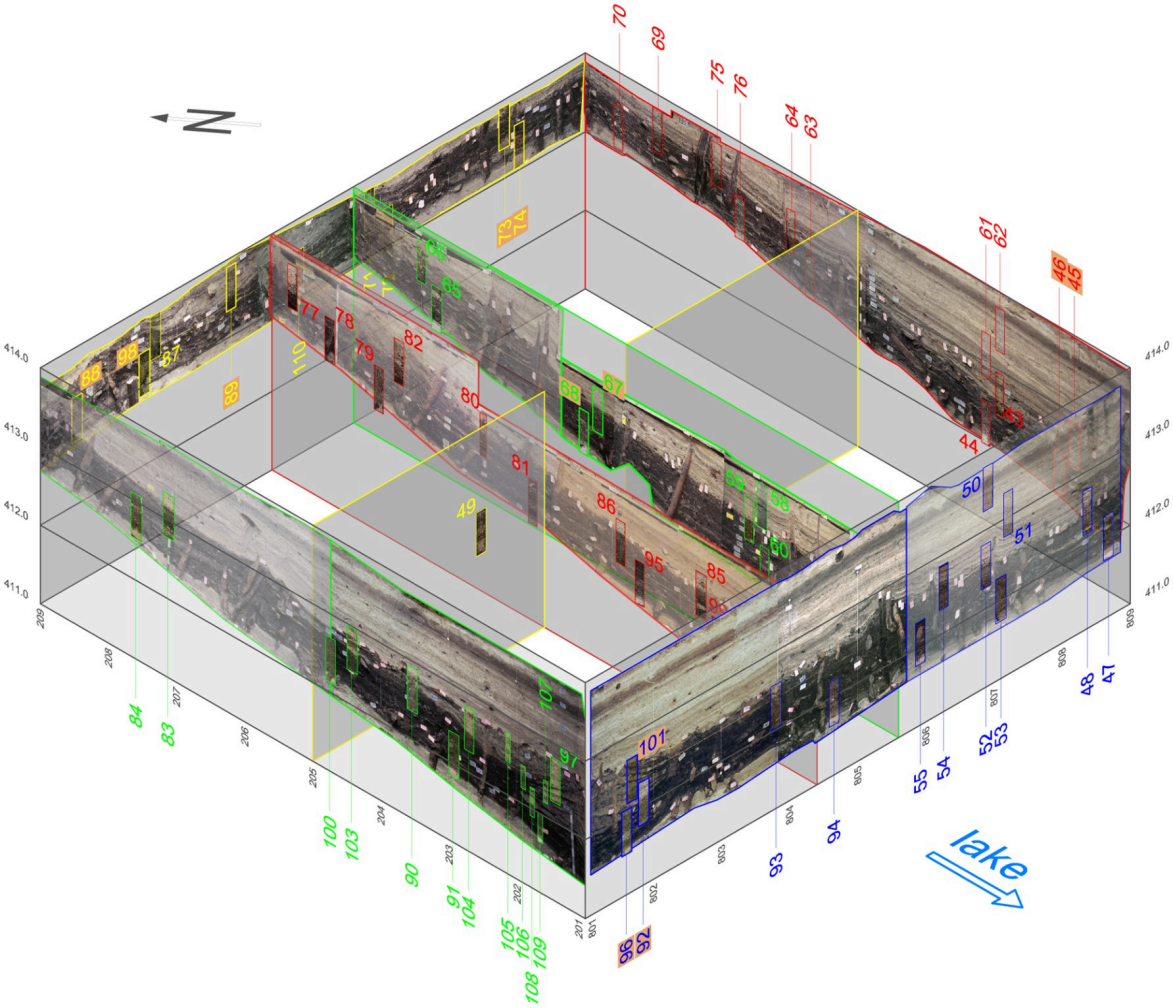
Position of the monolith columns collected at Zug-Riedmatt labelled by coloured numbers according to trench section. The five monolith sequences (12 columns in total) serving for the analysis are in orange (©ADA Zug). Easting, northing and elevation values are given in metres.

The Late Neolithic wetland settlement of Zug-Riedmatt (Canton of Zug, Switzerland; Fig 1) is along the northern shore of Lake Zug. Although today it is approximately 530 m from the shoreline, during the Neolithic it stood by the water within a bird-foot shaped delta formed at the mouth of the Lorze River (Fig 2). Its layers of occupation, up to 1.3 m thick (Fig 5), are very well preserved (sealed beneath 6 m of limnic and fluvial deposits) due to being constantly below the groundwater level. It is dated to between 3200 and 3050 cal BC (Horgen Culture) ([9]).

**Fig 6:**
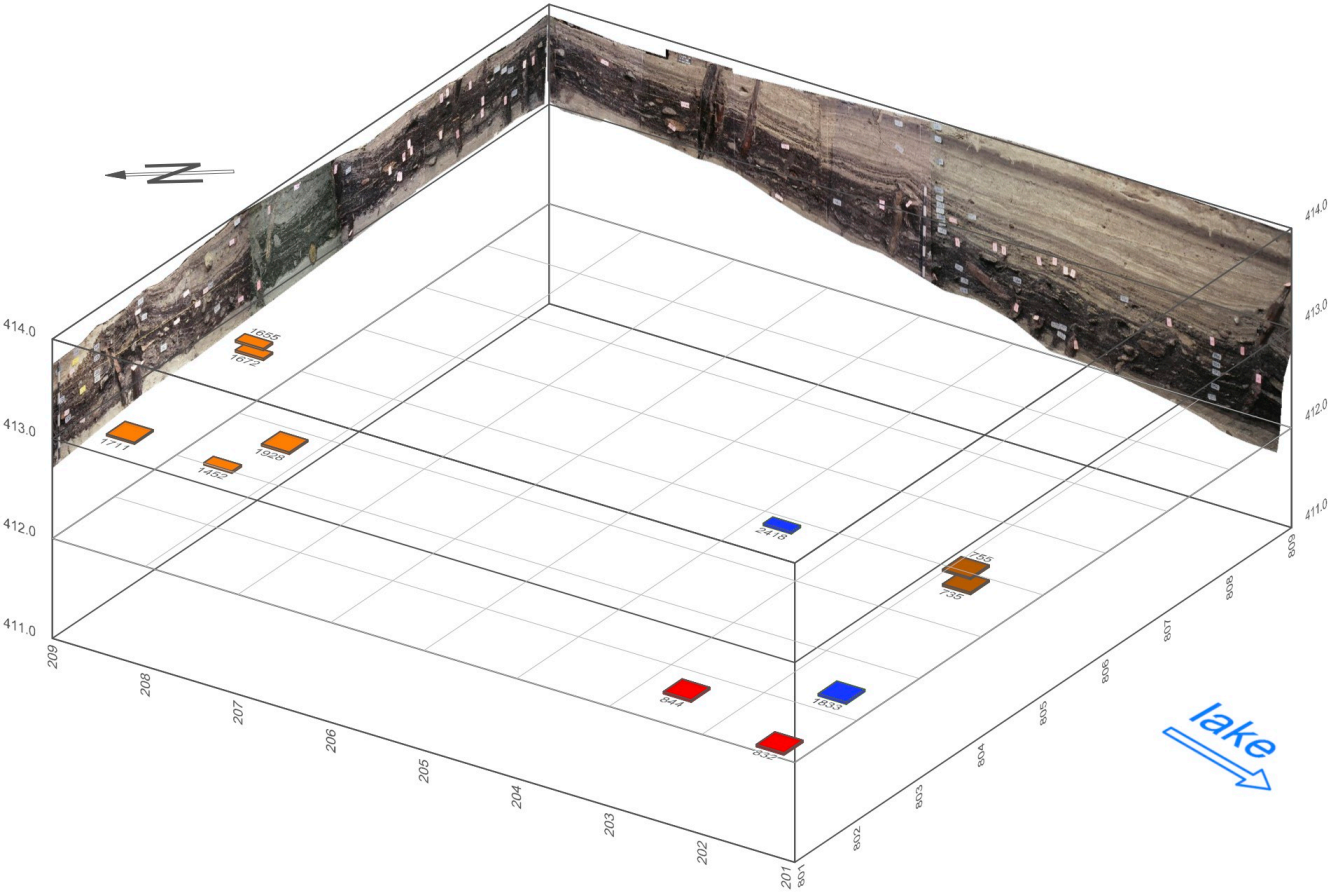
Position of the 11 bulk samples collected at Zug-Riedmatt (©ADA Zug). Samples from unit 3 in blue, unit 5 in orange, unit 6 in red and unit 8 in brown. Easting, northing and elevation values are given in metres.

According to a core drilling survey, the site extends over a surface of approximately 2500 m^2^. A small rescue excavation (64 m^2^) carried out in 2008 included a vast programme of soil samplings consisting of 110 monolith columns (usually 56×12×10 cm) collected from the cleaned profiles (Fig 5) and 607 large-volume bulk samplings (Fig 6).

The occupation of the settlement embedded in micrite (‘lake marl’) layers consists mostly of organic settlement layers in a sequence likewise containing organic micrite and loam (Fig 5, Table 1). The stratigraphy is subdivided into units comprising both anthropogenic and natural deposits (see Table 1). Units 3, 6 and 8 correspond to organic settlement layers while unit 11, predominantly loam, probably equates with another highly deteriorated settlement layer (hence not analysed in detail in this study). Unit 5, a loamy sediment rich in moss, red deer bones, fish scales (labelled the ‘bone midden’), on the contrary, formed part of this study. This unit belongs to the settlement layer comprising organic unit 3 (Table 1) and is worth highlighting due to its high content of waste from what appears to be a very brief timeframe ([12]).

The northern shore of Lake Zug is dominated by Gleysols and marsh soil types capable of retaining considerable volumes of water ([35]). The site was in an area that is poorly suited for arable farming. However, crop plants could have been grown in fields beyond the settlement such as in the fertile soils near Cham ([36]) or even farther along Lake Zug’s western coast in zones easily attained by boat [37].

### 2.2 Methods

#### 2.2.1 Archaeobotanical analyses

An earlier research project focusing on taphonomy (SNSF, project no. 149679) examined 197 small-volume samples from five continuous monolith columns of Zug-Riedmatt (see Fig 5, nos. 45–46; 67–68; 73–74; 88–89; 92, 96, and 101 marked in orange). The intention of the monolith sampling was to obtain lake-land and shore-parallel transects covering four sections of the excavated area. All five monolith sequences covered practically all of the layers between the micrite levels (‘lake marl’) ([38]; see also [8], 47). The task of separating the samples was undertaken in the laboratory so as to guarantee not mixing materials of one unit with another.

The present study only focuses on the phase of occupation, that is, the three organic units and the ‘bone midden’. Hence it only resorted to the monolith samples of these units for comparison. This reduced the number of small-volume monolith samples to 110, which can be broken down as follows: 15 from unit 3, 16 from unit 5, 34 from unit 6 and 45 from unit 8 (Table 2).

**Table 2:** Number and volume of the different monolith and bulk samples of units U3, U5, U6 and U8 serving for the analyses.

	Monolith		Bulk	
Unit	Number of samples	Total volume (L)	Number of samples	Total volume (L)
U3	15	5.64	2	12
U5	16	8.68	5	24
U6	34	10.13	2	10.5
U8	45	17.82	2	11

Since the main goal of the current study is to offer a palaeoeconomic evaluation, 11 large-volume bulk samples from the occupation layer were examined to enable their comparison with the monolith samples (Table 2). Two each were from predominantly organic units 3, 6 and 8, whereas the remaining five were from loamy unit 5 (‘bone midden’). Bulk samples often pose difficulties as archaeological units are not defined in the same detail during fieldwork as in the laboratory and may therefore be mixed with materials from other units. The bulk samples taken into account here were nonetheless carefully selected in order to ensure they correspond with the above-mentioned units. It is particular crucial in the case of multi-phased settlements to assure the stratigraphic validity of bulk samples before sieving. As certain samples at Zug-Riedmatt were erroneously ascribed to an incorrect unit, only those of unquestionable provenance were retained leading to a reduction to only a few per unit.

As the archaeological evaluation of Zug-Riedmatt has yet to be completed, this analysis currently lacks data as to the exact position of the samples, meaning it is not possible at the moment to determine whether they correspond to the settlement’s periphery or centre, or their position with regard to the structures (inside/outside or underneath). It is for this reason that this analysis focuses on the changes taking place between settlement phases.

The samples were sieved by the wash-over method ([14, 39, 40]). Most were frozen prior to sieving, a procedure that leads to a more gentle disintegration of the sediment concretions ([41]). Mesh size ranged from 4, 2 and 0.35 mm. Volumes were measured using the classical volume ([42]). A Leica/Wild M3Z stereo microscope (magnification 6.5-40x) served to sort and quantify the seed and fruit remains. Other macroscopic elements such as leaves, gastropods etc. were semiquantified. The smaller sieving fractions were often subsampled.

The seed reference collection of the Department of Environmental Sciences, University of Basel (IPAS) and specialised literature (e.g., [43, 44]) served to identify the plant remains. They were then quantified so as to roughly obtain a Minimum Number of Individuals (MNI) ([3]) before being recorded by the programme ArboDat (©Kreuz and Schäfer). Their classification into ecological groups was based on phytosociological and botanical literature ([45, 46]; www.infoflora.ch). Finally, the scientific nomenclature adheres to that of National Data and Information Centre of the Swiss Flora (www.infoflora.ch).

Comparisons of density (remains/litre, r/l) and numbers of plant taxa were then carried out between the monolith and bulk samples of the same units. The Shannon Index of Diversity (H_s_ = -∑(p_i_(lnp_i_)), Shannon Index of Evenness (E = H_s_/ln(S)) and Simpson’s Index of Diversity (1-D; D = ∑n_i_(n_i_-1)/N(N-1)) to measure heterogeneity were calculated by means of the PAST software ([47]). These indices combining taxa quantity with the abundance of each taxon commonly serve in ecology research to measure the heterogeneity within an assemblage. Although there are several aspects that differ among the archaeological and biological samples, the indices have been successfully applied to archaeobiological research (e.g., [31, 48]).

A Correspondence Analysis (CA) was likewise carried out through PAST by resorting to density values of all taxa defined as cultivated or gathered fruits (charred as well as waterlogged and all types of remains). Furthermore, proportions were calculated for the most relevant taxa of the cultivated and gathered fruits. Only one type of (waterlogged) find was considered per taxon (e.g., seeds, not capsules, served to calculate the proportion of flax).

The method applied to calculate calorie intake was drawn mostly from [4] (see Table 2 of [4], obtained from several bibliographical sources: [3] and their references [49, 50]). The additional species are referenced by [51–56] (see S1 Table). In fact, calculating calorie intake is not straightforward as it only is a rough approximation of the real values. Finally, the calculations of density and proportion were undertaken with RStudio 4.0.5 ([57]).

#### 2.2.2 Chronology

Despite intensive research by the SNSF taphonomy research project cited above, the chronology of the different settlement layers remains vague. Wetland site chronology is most often defined by dendrochronological analyses of elements of construction. However, this method for the settlements along the shores of Lake Zug such as Riedmatt is complicated by several factors, notably that the residents resorted to relatively short-lived riparian forest species and coppiced trees for construction ([9]). As only the last layer of Zug-Riedmatt benefitted from dendro-dating (3056–3045 cal BC, Bleicher, pers. com.), a series of radiocarbon datings of cultivated short-lived plant macrofossils were undertaken to determine the timeframes of the different Neolithic occupations (S2 Table) and complement earlier datings of the intermediate, unoccupied layers (Table 3). Two different models deriving from both OxCal v4.4.2 ([58]) and the atmospheric curve IntCal20 ([59]) were developed to define the site’s chronological timeframe.

**Table 3:** AMS radiocarbon datings of waterlogged finds from Zug-Riedmatt (nd = undetermined).

Lab. code	14C age BP	SD	cal BC (2σ)	Sample	Species	Period	Stratigraphic association	Reference
ETH-78816	4516	35	-3361–3098	moss	nd	Late Neolithic (*Horgen*)	Unit 1	[9]
ETH-78817	4546	27	-3370–3103	needles	*Abies* sp.	Late Neolithic (*Horgen*)	Unit 2	[9]
ETH-110809	4644	74	-3633–3105	chaff	*Triticum aestivum/durum/turgidum*	Late Neolithic (*Horgen*)	Unit 3	this paper—AgriChange project
ETH-78818	4583	26	-3495–3116	fruit shell	*Corylus avellana*	Late Neolithic (*Horgen*)	Unit 3	[9]
ETH-78819	4477	26	-3340–3031	pericarp	*Malus/Pyrus*	Late Neolithic (*Horgen*)	Unit 4	[9]
ETH-88881	4558	25	-3482–3105	seed	*Linum usitatissimum*	Late Neolithic (*Horgen*)	Unit 5	this paper—AgriChange project
ETH-88877	4539	24	-3366–3103	seed	*Malus sylvestris*	Late Neolithic (*Horgen*)	Unit 5	this paper—AgriChange project
ETH-74496	4513	23	-3354–3101	fruit shell	*Corylus avellana*	Late Neolithic (*Horgen*)	Unit 5	[9]
ETH-88879	4506	25	-3351–3099	seed	*Triticum aestivum/durum/turgidum*	Late Neolithic (*Horgen*)	Unit 6	this paper—AgriChange project
ETH-74497	4475	23	-3337–3032	fruit shell	*Corylus avellana*	Late Neolithic (*Horgen*)	Unit 6	[9]
ETH-74498	4470	23	-3336–3029	fruit shell	*Corylus avellana*	Late Neolithic (*Horgen*)	Unit 7	[9]
ETH-74499	4473	23	-3336–3031	seed	*Prunus spinosa*	Late Neolithic (*Horgen*)	Unit 7/8?	[9]
ETH-88878	4555	25	-3371–3103	seed	*Triticum dicoccum*	Late Neolithic (*Horgen*)	Unit 8	this paper—AgriChange project
ETH-74500	4488	24	-3344–3092	fruit shell	*Corylus avellana*	Late Neolithic (*Horgen*)	Unit 8	[9]
ETH-74502	4429	24	-3322–2928	axe shaft, core of branch	*Quercus* sp.	Late Neolithic (*Horgen*)	Unit 8	[9]
ETH-74501	4467	24	-3335–3028	fruit shell	*Corylus avellana*	Late Neolithic (*Horgen*)	Unit 8/9	[9]
ETH-88880	4539	25	-3366–3103	seed	*Triticum aestivum/durum/turgidum*	Late Neolithic (*Horgen*)	Unit 9	this paper—AgriChange project
ETH-78820	4498	26	-3346–3097	fruit shell	*Corylus avellana*	Late Neolithic (*Horgen*)	Unit 9	[9]
ETH-78821	4450	26	-3335–3012	fruit shell	*Corylus avellana*	Late Neolithic (*Horgen*)	Unit 10	[9]
ETH-84424.1.1	4502	22	-3346–3099	wood	*Abies alba*	Late Neolithic (*Horgen*)	Unit 11/12	Huber et al. 2020 [63]
ETH-84425.1.1	4443	22	-3330–2937	wood	*Abies alba*	Late Neolithic (*Horgen*)	Unit 11/12	Huber et al. 2020 [63]
ETH-78822	4439	26	-3330–2931	fruit shell	*Corylus avellana*	Late Neolithic (*Horgen*)	Unit 11/12	[9]
ETH-78824	4485	35	-3348–3031	needles	*Abies sp*.	Late Neolithic (*Horgen*)	Unit 14	[9]
ETH-78823	4482	27	-3341–3034	fruit shell	*Corylus avellana*	Late Neolithic (*Horgen*)	Unit 14	[9]
ETH-71517	3158	23	-1499–1397	branchlet/root	nd	Late Middle Bronze Age	above Unit 14	Huber et al. 2020 [63]
ETH-71516	3114	22	-1440–1299	seed	*Schoenoplectus* sp.	Late Middle Bronze Age	above Unit 14	Huber et al. 2020 [63]
ETH-71518	3103	22	-1430–1293	seed	*Alnus sp*.	Late Middle Bronze Age	above Unit 14	Huber et al. 2020 [63]

The Kernel Density Estimation model (KDE) was based on datings of both settlement layers and the layers of abandonment between them. This method allows interpreting the probability distribution of the datings of the different units within a group in a way that retains the signal and suppresses the noise of the precision of the atmospheric curve used to calibrate the raw dates ([60]).

As note above, the different radiocarbon dates were carried out on a succession of clearly stratified units. This succession of datings with a start and end boundary ([58]) allowed generating a 11-phase contiguous Bayesian model serving to determine the individual as well as the total duration of the occupation. The model was restricted to a range between 3250 and 3050 cal BC ([58]), a timeframe garnered by comparing elements of Zug-Riedmatt’s material culture (pottery and deer antler artefacts) with those of nearby dendro-dated settlements (for this chronotypology see e.g., [61, 62]). A terminus post quem of 3250 cal BC was thus established for the datings subsequent to the outset of unit 3 and a *terminus ante quem* of 3050 cal BC for the end of the last phase.

#### 2.2.3 Seasonal climate and crop growing conditions—The Δ^13^C values of cereals

The determination of the level of concentration and isotope composition of carbon was carried out with a ThermoFisher Flash-EA 1112 with a Conflo IV interface coupled to a ThermoFisher Delta V isotope ratio mass spectrometer (IRMS). Samples were fired in the presence of O^2^ in an oxidation column at 1020°C. The gases of the combustion were then subjected to a reduction column (650°C) yielding N^2^ and CO^2^ which were separated chromatographically and transferred to the IRMS via an open split for on-line isotope measurements. The isotope ratios were reported in the conventional δ-notation with respect to the Vienna Pee Dee Belemnite (V-PDB) standard. The system was calibrated with NBS22 (δ^13^C = -30.03) and IAEA CH-6 (δ^13^C = -10.46). The reproducibility of the measurements was greater than 0.2‰.

The carbon isotope of the chaff and the estimated δ^13^C of the atmospheric CO2 served to calculate the Δ^13^C values of 69 uncharred chaff samples (individual spikelet forks or rachis segments; Table 4) of naked wheat (*Triticum aestivum/durum/turgidum*) and emmer wheat (*Triticum dicoccon*) from units 3, 5, 6 and 8 (S3 Table).

**Table 4:** Δ^13^C values of uncharred chaff samples from units 3, 5, 6 and 8 dated to the Late Neolithic (Horgen Culture).

Layer	Species	δ13C (VPDB)	Δ13C
U3	*Triticum dicoccum*	-30.83	25.25
U3	*Triticum dicoccum*	-30.27	24.66
U3	*Triticum dicoccum*	-29.57	23.92
U3	*Triticum dicoccum*	-29.50	23.85
U3	*Triticum dicoccum*	-31.28	25.73
U3	*Triticum dicoccum*	-31.32	25.77
U3	*Triticum dicoccum*	-31.01	25.44
U3	*Triticum dicoccum*	-30.68	25.10
U3	*Triticum aestivum/durum/turgidum*	-30.98	25.41
U3	*Triticum aestivum/durum/turgidum*	-30.74	25.16
U3	*Triticum aestivum/durum/turgidum*	-29.48	23.83
U3	*Triticum aestivum/durum/turgidum*	-31.37	25.82
U3	*Triticum aestivum/durum/turgidum*	-31.24	25.69
U3	*Triticum aestivum/durum/turgidum*	-31.08	25.52
U3	*Triticum aestivum/durum/turgidum*	-29.46	23.80
U5	*Triticum dicoccum*	-30.39	24.78
U5	*Triticum dicoccum*	-30.96	25.39
U5	*Triticum dicoccum*	-29.94	24.31
U5	*Triticum dicoccum*	-31.06	25.50
U5	*Triticum dicoccum*	-30.86	25.28
U5	*Triticum dicoccum*	-30.44	24.84
U5	*Triticum dicoccum*	-30.02	24.39
U5	*Triticum dicoccum*	-31.68	26.16
U5	*Triticum dicoccum*	-29.85	24.22
U5	*Triticum aestivum/durum/turgidum*	-30.88	25.31
U5	*Triticum aestivum/durum/turgidum*	-31.67	26.14
U5	*Triticum aestivum/durum/turgidum*	-30.43	24.83
U5	*Triticum aestivum/durum/turgidum*	-31.56	26.03
U5	*Triticum aestivum/durum/turgidum*	-30.41	24.81
U5	*Triticum aestivum/durum/turgidum*	-30.30	24.70
U6	*Triticum dicoccum*	-31.02	25.46
U6	*Triticum dicoccum*	-30.23	24.62
U6	*Triticum dicoccum*	-31.71	26.18
U6	*Triticum dicoccum*	-30.71	25.12
U6	*Triticum dicoccum*	-29.88	24.25
U6	*Triticum dicoccum*	-31.02	25.45
U6	*Triticum dicoccum*	-30.43	24.83
U6	*Triticum dicoccum*	-30.21	24.59
U6	*Triticum aestivum/durum/turgidum*	-31.24	25.69
U6	*Triticum aestivum/durum/turgidum*	-30.17	24.55
U6	*Triticum aestivum/durum/turgidum*	-30.40	24.80
U6	*Triticum aestivum/durum/turgidum*	-30.69	25.11
U6	*Triticum aestivum/durum/turgidum*	-30.62	25.03
U6	*Triticum aestivum/durum/turgidum*	-31.06	25.49
U6	*Triticum aestivum/durum/turgidum*	-30.07	24.45
U6	*Triticum aestivum/durum/turgidum*	-31.60	26.07
U6	*Triticum aestivum/durum/turgidum*	-29.65	24.01
U6	*Triticum dicoccum*	-31.40	25.86
U6	*Triticum dicoccum*	-30.53	23.46
U8	*Triticum dicoccum*	-29.88	24.25
U8	*Triticum dicoccum*	-29.61	23.96
U8	*Triticum dicoccum*	-30.32	24.71
U8	*Triticum dicoccum*	-30.44	24.84
U8	*Triticum dicoccum*	-30.00	24.38
U8	*Triticum dicoccum*	-30.46	24.86
U8	*Triticum dicoccum*	-30.74	25.16
U8	*Triticum dicoccum*	-30.24	24.63
U8	*Triticum dicoccum*	-30.50	24.91
U8	*Triticum dicoccum*	-29.26	23.60
U8	*Triticum aestivum/durum/turgidum*	-30.35	24.74
U8	*Triticum aestivum/durum/turgidum*	-30.25	24.64
U8	*Triticum aestivum/durum/turgidum*	-31.88	26.36
U8	*Triticum aestivum/durum/turgidum*	-30.97	25.40
U8	*Triticum aestivum/durum/turgidum*	-30.58	24.99
U8	*Triticum aestivum/durum/turgidum*	-31.29	25.74
U8	*Triticum aestivum/durum/turgidum*	-30.43	24.83
U8	*Triticum aestivum/durum/turgidum*	-30.05	24.42
U8	*Triticum aestivum/durum/turgidum*	-31.17	24.07
U8	*Triticum aestivum/durum/turgidum*	-30.82	23.74

Plants normally react to a reduction or increase in water availability through the closing or opening of their stomata. Hence δ^13^C of plant tissues is a good indicator of water status when these tissues are formed ([64]). Carbon isotope discrimination (Δ^13^C) is a tool that may offer reliable data on water availability for ancient crops. The Δ^13^C values may reflect climatic fluctuations, but in the case of crops they may also reflect the effect of certain agronomic practices such as planting in naturally moist soils on the water status of crops (e.g. [65, 66]). Other factors such as altitude and temperature, and if growing locations greatly differ ([67, 68]), can influence the results of these analyses. Determining the Δ^13^C values of the samples of this study consisted of applying the following formula based on δ^13^C_air_ and plant carbon isotope composition ([69]) from the CU-INSTAAR/NOAA-CMDL database ([66], http://web.udl.es/usuaris/x3845331/AIRCO2_LOESS.xls):

$$\Delta 13C=\frac{\delta^{13}C_{air}-\delta^{13}C_{plant}}{\left(1+\frac{\delta^{13}C_{plant}}{1000}\right)}$$

Obtaining the δ^13^C_air_ mean and δ^13^C_air_ standard error necessary to calibrate the δ^13^C of the samples requires a dating range. The study thus resorted to the initial and final chronological boundaries indicated by the Bayesian model of the different units (see section 3.5). As there is no evidence of ancient artificial irrigation, the Δ^13^C values must be interpreted as a result of the interaction of the characteristics of the soil with precipitation during the period of formation of the chaff. All graphs and statistical tests of this data were generated by the RStudio version 4.0.5 application (2021 [57]).

## 3. Results

### 3.1 General findings

The current research combined with that of the previous project led to the sorting and quantifying of 139,430 botanical macroremains which were subsequently classified into 195 different taxa. Over 99% were preserved in a subfossil (uncharred) state. The most common crop plant remains throughout all the units were the seeds of opium poppy (*Papaver somniferum*), seeds and parts of capsules of flax (*Linum usitatissimum*) and the subfossil chaff of a variety of cereals, mostly emmer wheat (*Triticum dicoccon*), tetraploid naked wheat (*Triticum durum/turgidum*) and barley (*Hordeum vulgare*). Pea pod fragments (*Pisum sativum*) were also frequent. The most common remains of gathered fruits were seeds of wild strawberry (*Fragaria vesca*), raspberry (*Rubus idaeus*), blackbery (*Rubus fruticosus*), seeds and pericarps of crab apple (*Malus sylvestris*), shells of hazelnut (*Corylus avellana*) and pericarps of acorn (*Quercus sp*.). Seeds of winter cherry (*Physalis alkekengi*), rose hip (*Rosa sp*.) and beechnut (*Fagus sylvatica*) were less common. The ecological groups best-represented by both the monolith and bulk sampling techniques were woodland plants (mostly needles of silver fir, *Abies alba*), summer crop annual weeds and annual ruderals (often vervain, *Verbena officinalis* and thyme-leaved sandwort, *Arenaria sepyllifolia*), wet grassland plants (mostly greater plantain, *Plantago major*, which actually has a broad ecological amplitude) and woodland clearing edge, hedge and bush plants (see S4 Table for individual species, preservation and type of remains).

**Fig 7:**
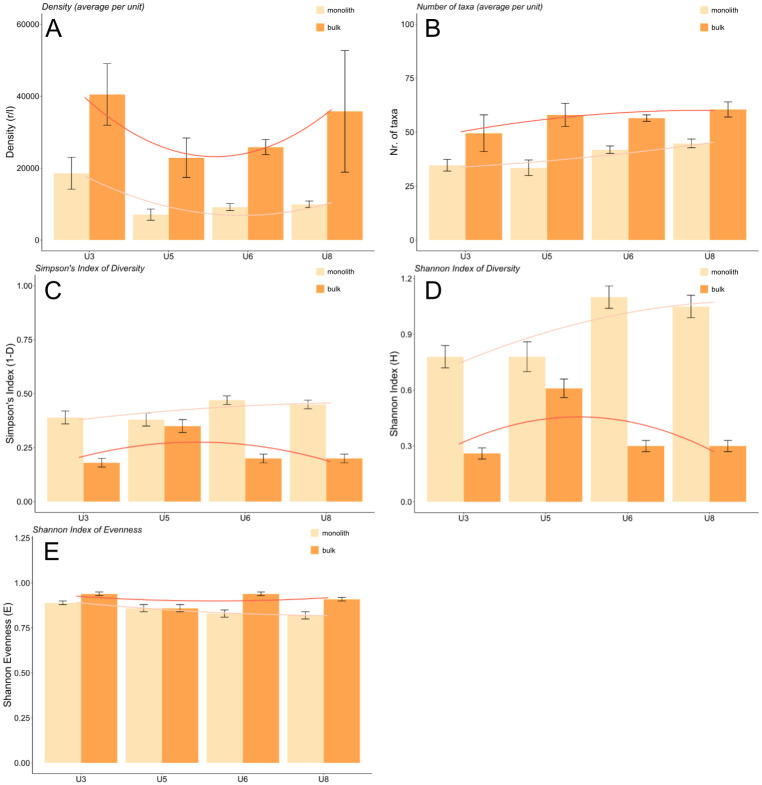
Plant assemblage composition (mean ±standard error) A) Density, B) number of taxa, C) Simpson’s Index of Diversity, D) Shannon Index of Diversity, E) Shannon Index of Evenness.

The mean volume of the 11 large-volume bulk samples was 5.2 litres (max. 7.0; min. 4.0 l) and the mean density was 28,967.0 r/l (max. 52,706.2, min. 9,019.3 r/l). The mean volume of the 110 small-volume monolith samples, in turn, is 0.4 litres (max. 3.5; min. 0.05 l) and the mean density is 10,468.8 r/l (max. 68,753.3, min. 157.3 r/l). Mean density values and mean taxa number (per sample) were always greater among the bulk samples (Fig 7A and 7B). More taxa were identified in the bulk samples and in greater numbers per sample (although the total number of taxa among the monolith samples was higher due to having analysed a greater number). All the ecological groups and plant taxa of Zug-Riedmatt were not affected equally.

The ecological groupings of reed bed and sedge swamp plants, undetermined wetland plants, winter crop annual and flax weeds, perennial ruderals, potentially cultivated plants (*Apium graveolens*, *Anethum graveolens*) revealed higher mean densities among all the monolith samples of the tested units. The ecological grouping of woodland plants, as well as that of cereals, pulses and oil and fibre plants, on the contrary, yielded higher mean densities among the bulk samples of all tested units.

Furthermore, heterogeneity values (Simpson’s Index of Diversity, Shannon Index of Diversity) were higher among the monolith samples. The diversity of their assemblage is therefore greater despite their smaller sample size (Fig 7C and 7D). The smallest differences between the heterogeneity values of the monolith and bulk samplings correspond to unit 5.

The values of evenness (Shannon Index of Evenness) were generally high, suggesting an equal abundance of all species (Fig 7E). Values of the bulk samples were higher in three cases, indicating that the degree of diversity and taxonomic distribution detected in the bulk samples is somewhat less accurate than that of the monolith samples.

### 3.2 Correspondence Analysis (CA)

**Fig 8:**
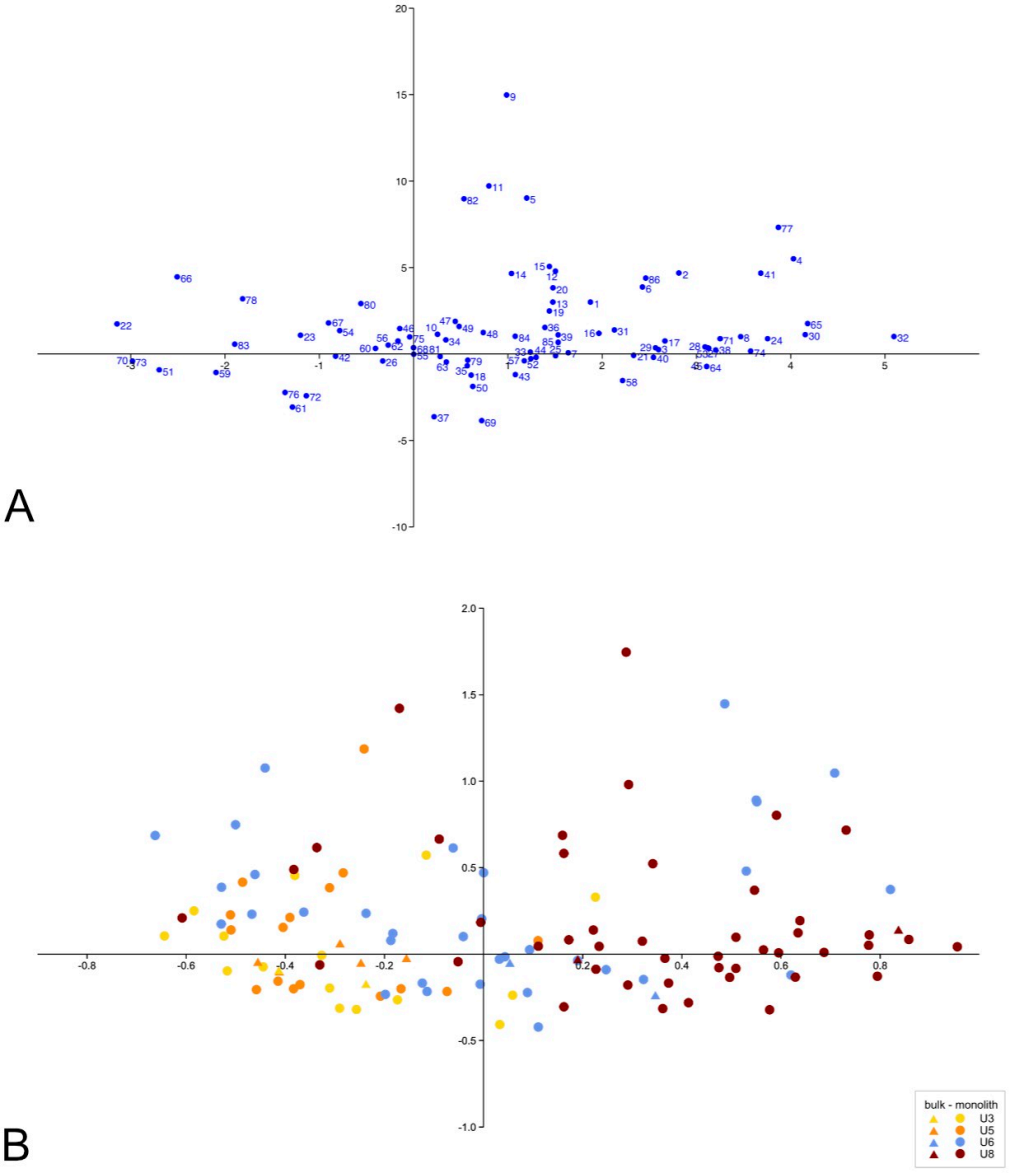
Graph depicting the findings of the Correspondence Analysis based on the values of density of all cultivated and gathered taxa (the first two dimensions are listed, added percentage variation is 32.4%). **A:** Columns (numbers equate with taxa, see S4 Table for Scientific names, types of remains and preservation); **B:** Rows (triangles = bulk samples; circles = monolith samples).

The Correspondence Analysis of cultivated plants and gathered fruits (86 waterlogged and charred taxa, see S4 Table for taxa codes) suggest that it is possible to discriminate (albeit roughly) between the samples from different units (Fig 8). Those of units 3 and 5 fall into the negative field of the y-axis, linked notably to opium poppy, winter cherry, wild celery, etc. Those of unit 8, in turn, fall on the positive side of the y-axis, associated with many charred plants such as pea (different types and states preservation), naked wheat chaff, etc. There are few exceptions such as those of unit 6 that are distributed over the whole of the chart. Hence the samples from earlier units 3 and 5 appear to clearly set themselves apart from those of later unit 8. Samples from unit 6 spread over the whole chart can be partly associated with those from all units indicating that they represent an amalgam of both early and later units. The grouping of bulk and monolith samples from identical units rather than of all the bulk samples and all monolith samples together implies that differences between units are much greater than those between sample techniques.

### 3.3 Crop plants

#### 3.3.1 Crop plant density

**Fig 9:**
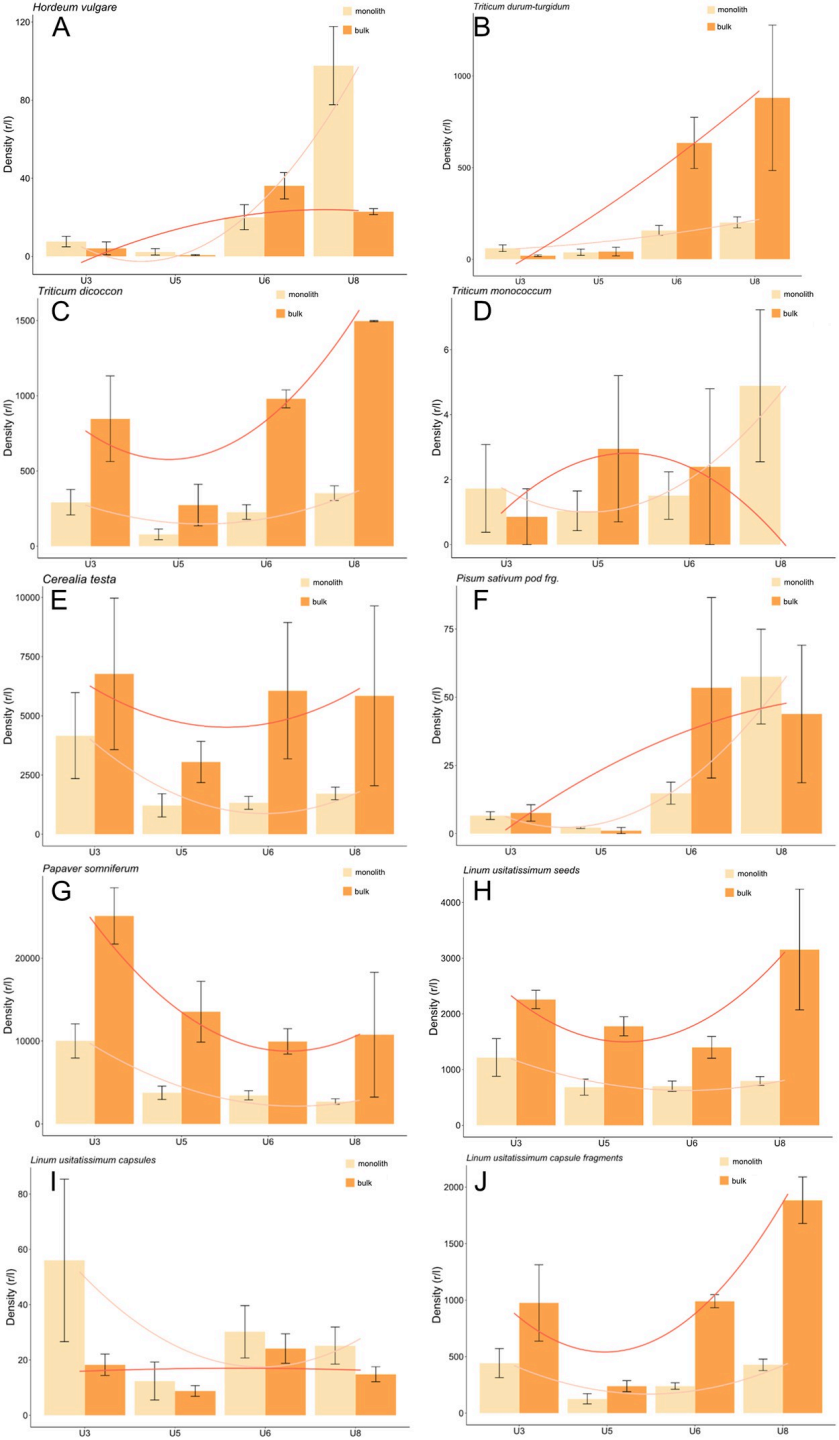
A-J Density of waterlogged crop plants (mean ±standard error).

The density of subfossil barley and naked wheat chaff increased over time among the samples collected by both the monolith and bulk techniques (Fig 9A and 9B). Subfossil emmer wheat chaff, although also yielding a great density in unit 8, revealed much greater values than barley and naked wheat in units 3, 5 and 6 and is the most common cereal throughout all the settlement layers (Fig 9C). The density of the remains from both monolith and bulk samples matched for the most part, although the values of the monolith samples were comparatively higher for unit 3. Furthermore, einkorn wheat subfossil chaff revealed low densities in both monolith and bulk samplings and throughout all the units under study (Fig 9D).

The greatest densities of cereal grain testa fragments appeared in unit 3. While the density among the monolith samples decreased considerably in units 5, 6 and 8, the difference was not as pronounced among the samples collected through bulk sampling (Fig 9E).

**Fig 10:**
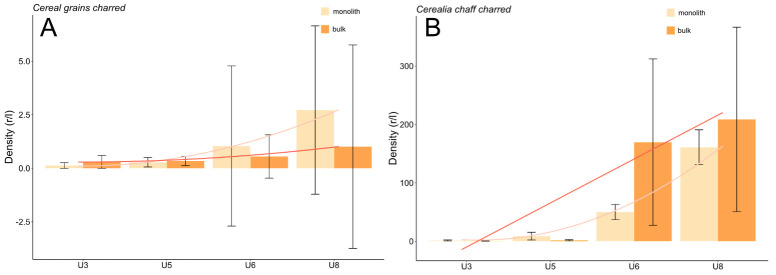
A, B Density of charred cereal grains and chaff (mean ±standard error).

The density of charred cereals remained very low in units 3 and 5 (grains and chaff; Fig 10A and 10B). The densities of both charred cereal grain and chaff increased in unit 6, a tendency that grew even more in unit 8 among both monolith and bulk samples. Moreover, despite a high standard error, charred cereal grains were more often found in monolith samples while the same appeared to be the case of chaff among the bulk samples.

The density of subfossil pea pod fragments also increased over time with greater frequency in units 6 and 8 (Fig 9F). Moreover, the waterlogged and charred pea seeds were exclusive to these two units.

The densities of subfossil poppy seeds, in turn, attained their highest values in unit 3 and decreased over time in both monolith and bulk samples (Fig 9G). Poppy capsules however, are represented in low density in unit 3 as well as in units 5 and 8.

The density of subfossil flax remains among both monolith and bulk samples differed more than that of other crop plants. While the highest density of all flax remains (seeds and capsule fragments) among the monolith samples was in unit 3, those of bulk samples were in unit 8 (except for the flax capsules of unit 6; Fig 9H–9J). These differences between units were not as pronounced as those of poppy.

#### 3.3.2 Crop plant proportions

**Fig 11:**
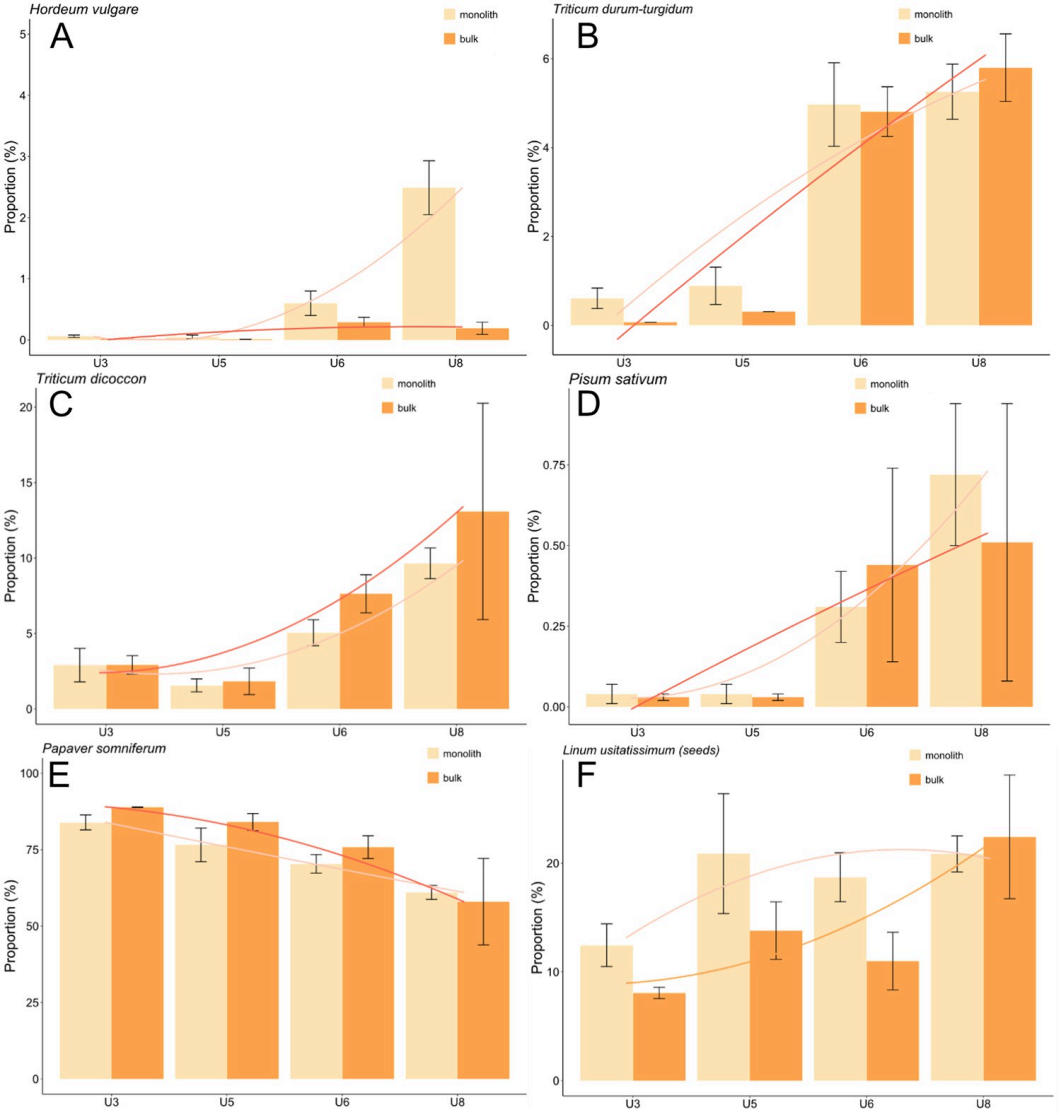
A-F Proportions of waterlogged selected crop plants (mean ±standard error).

The proportion (percentage based on density) of crop plants resembles that of all taxa (Fig 11A–11D) except for flax (Fig 11F). In short, the proportions of barley, naked wheat, emmer wheat and pea increase in unit 8, whereas opium poppy decreases from unit 3 to 8 (although still representing >50% of unit 8). This is the case for both monolith and bulk samples. Flax, revealing the highest proportions after opium poppy, differed the most between monolith and bulk samples, but unlike density, its proportions were highest among both monolith and bulk samples of unit 8 (although that of monolith samples was comparably high in unit 5).

#### 3.3.3 Crop plant caloric input

**Fig 12:**
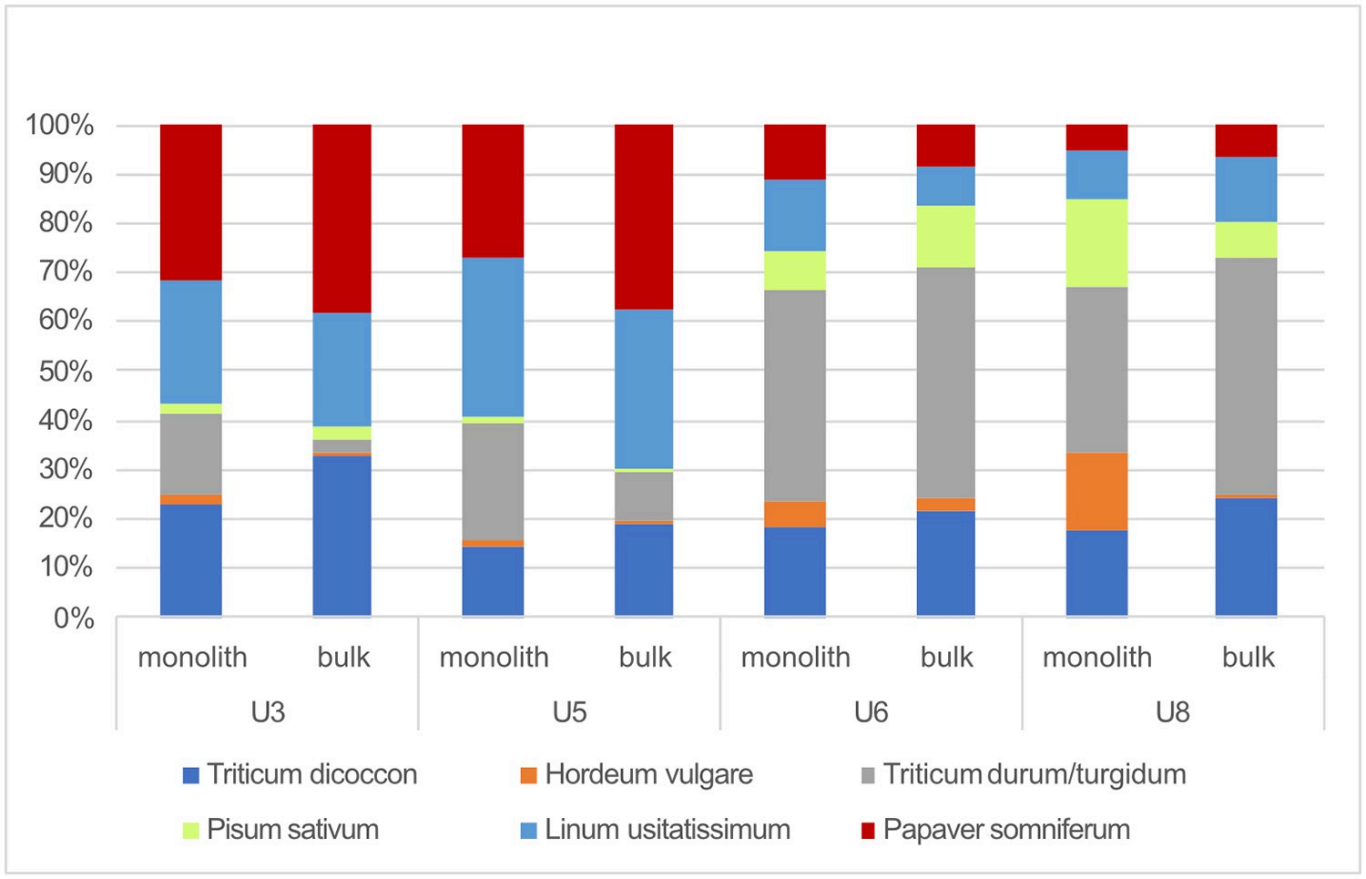
Caloric input from cultivated plants.

A comparison of units 3/5 and units 6/8 suggests that cultivars yielded differences of caloric input (Fig 12). The calories associated with the remains of opium poppy and flax of units 3/5 account for a much higher percentage among each of the sampling techniques. The percentages of naked wheat and pea of units 6/8 increased among both monolith and bulk samples. The percentage of barley also grew, in particular among the monolith samples. The caloric input from emmer wheat, in turn, was more or less constant throughout all units.

Only about 30–40% of the caloric input from the crop plants of units 3/5 stemmed from cereals. Units 6/8, in turn, revealed a growth of input from cereals to about 70%. However, when considering unidentified chaff (identified no further than *Triticum sp*. or Cerealia), which makes up around 50% of all chaff, the caloric input from cereals would presumably increase to about 45–60% in units 3/5 and >80% in units 6/8.

### 3.4 Gathered fruits

#### 3.4.1 Gathered fruit density

**Fig 13:**
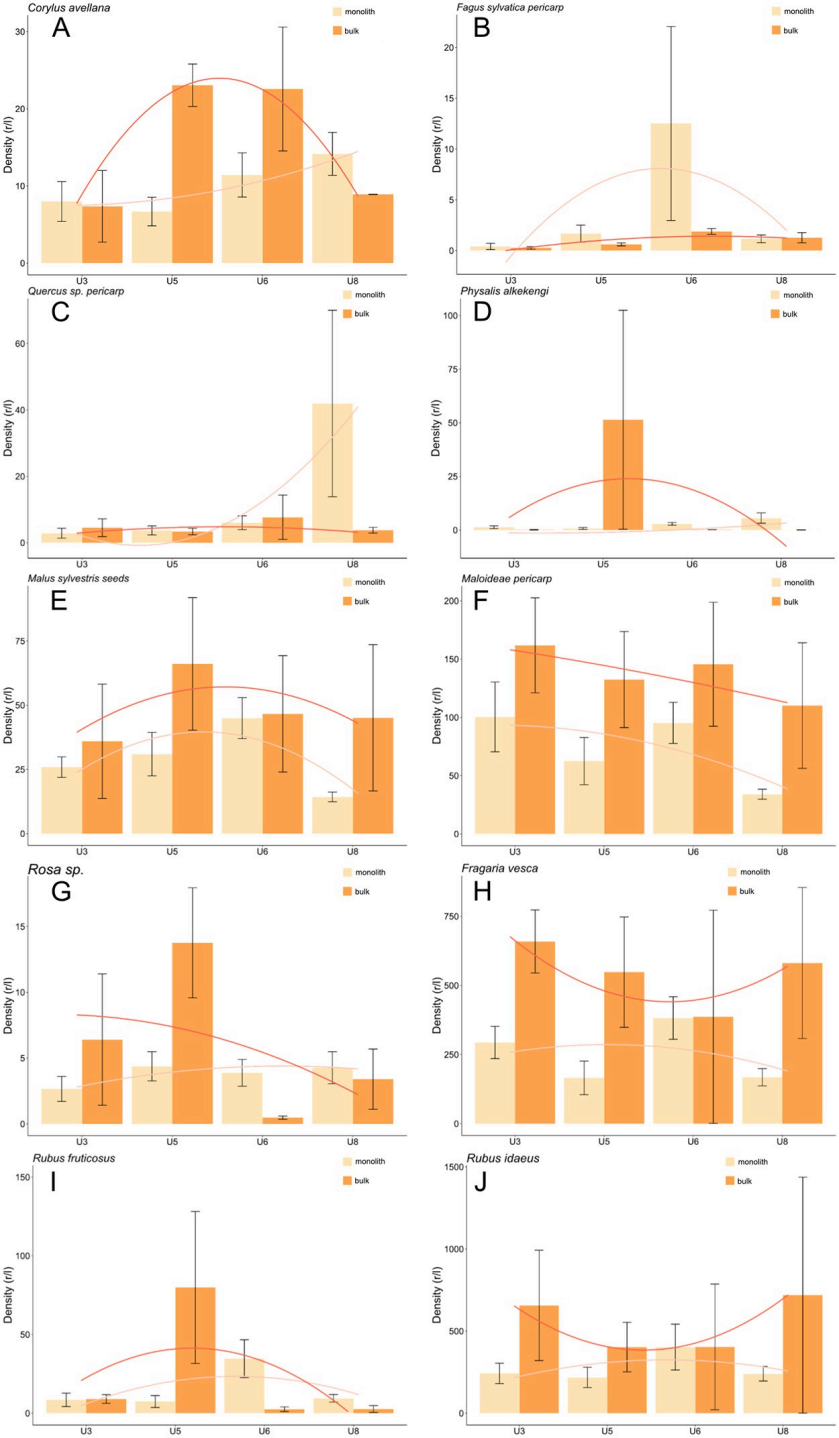
A-J Density of gathered waterlogged fruits (mean ±standard error).

The numbers between the waterlogged gathered fruit samples collected by monolith and bulk techniques differed more often than those of crop plants (Fig 13A–13J) rendering it difficult to define general trends. Bulk samples usually revealed higher gathered fruit densities. The most notable exceptions were the beech pericarps, rose and blackberry nutlets of unit 6 and the acorn pericarps and hazelnut shells of unit 8. Gathered fruits collected through monolith sampling more often attained higher densities in unit 6 and 8 while bulk samples more often yielded higher densities in unit 5.

**Fig 14:**
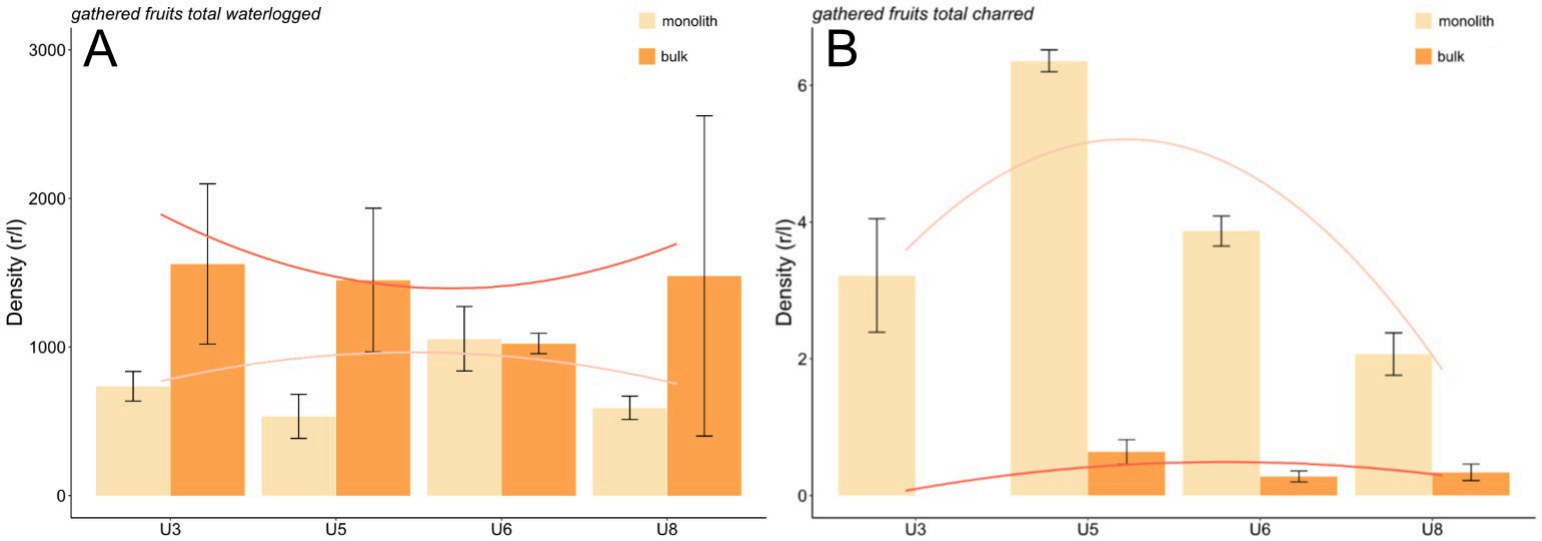
A, B Density of gathered fruits (summed density, waterlogged and charred preservation) (mean ±standard error).

Gathered fruit totals from bulk samplings revealed high levels of density among units 3, 5 and 8 (contrary to unit 6). The total from the monolith sampling, in turn, revealed the highest density in unit 6 (Fig 14A). However, the standard error among the bulk samples is quite large for units 3, 5 and especially 8.

The density of charred gathered fruits was low, never exceeding 1 r/l in the bulk samples, whereas its levels the monolith samples were slightly higher, attaining a maximum in unit 5 (Fig 14B).

#### 3.4.2 Gathered fruit proportions

**Fig 15:**
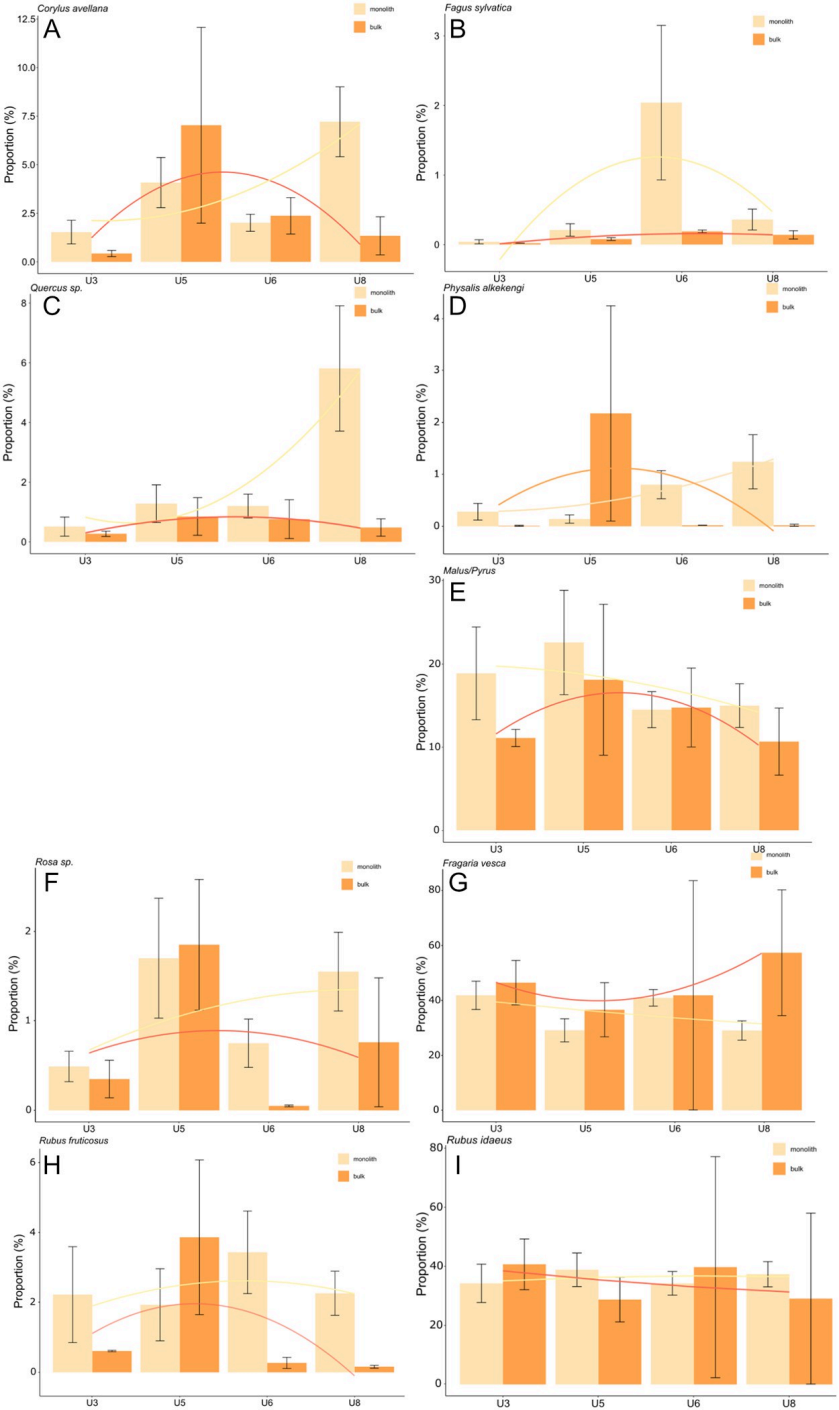
A-I Proportions of selected gathered fruits (waterlogged preservation) (mean ±standard error).

As in the case of density, the proportions of gathered fruits differed more often between the monolith and bulk samples than those of cultivated plants (Fig 15A–15I). These differences were highest among the taxa with lower proportions that peaked in only one sample type and unit (i.e., beech in the unit 6 monolith samples, Fig 15B, mostly due to sample 88.11 containing >300 r/l of beechnuts). Among the taxa represented by high proportions, the differences between the types of samples were not as great and did not change as much throughout the different units. The results are mostly comparable to those of the densities of the same taxa.

Gathered fruit collected in the monolith samples attained their highest proportions in units 5, 6 and 8 while those in the bulk samples often attained their highest densities in unit 5.

#### 3.4.3 Gathered fruit caloric input

**Fig 16:**
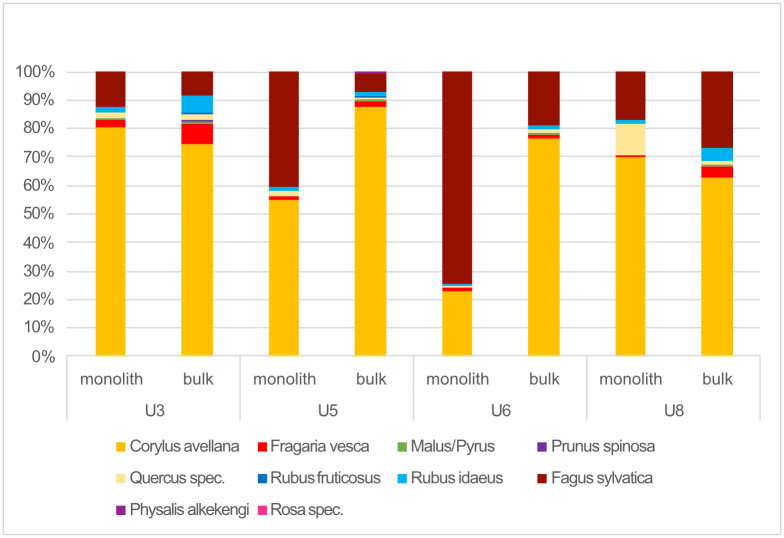
Caloric input based from gathered fruit samples.

As in the case of density and proportions, a comparison of the differences of caloric input potentially offered by gathered fruits points to great divergency between the monolith and bulk techniques (Fig 16). This mostly affected unit 5, and especially unit 6, where the monolith samples reveal more beechnuts than hazelnuts. The monolith samples of unit 8 reveal a higher caloric input of acorns than those of bulk samples. However, hazelnuts contributed the most to the levels of caloric input throughout all units and sample types (except, as noted, unit 6 of the monolith samples), followed by beechnuts. Moreover, apart from the acorns of the monolith samples of unit 8, no other gathered fruit contributed more than 10% of the caloric input to any unit.

### 3.5 Chronology

**Fig 17:**
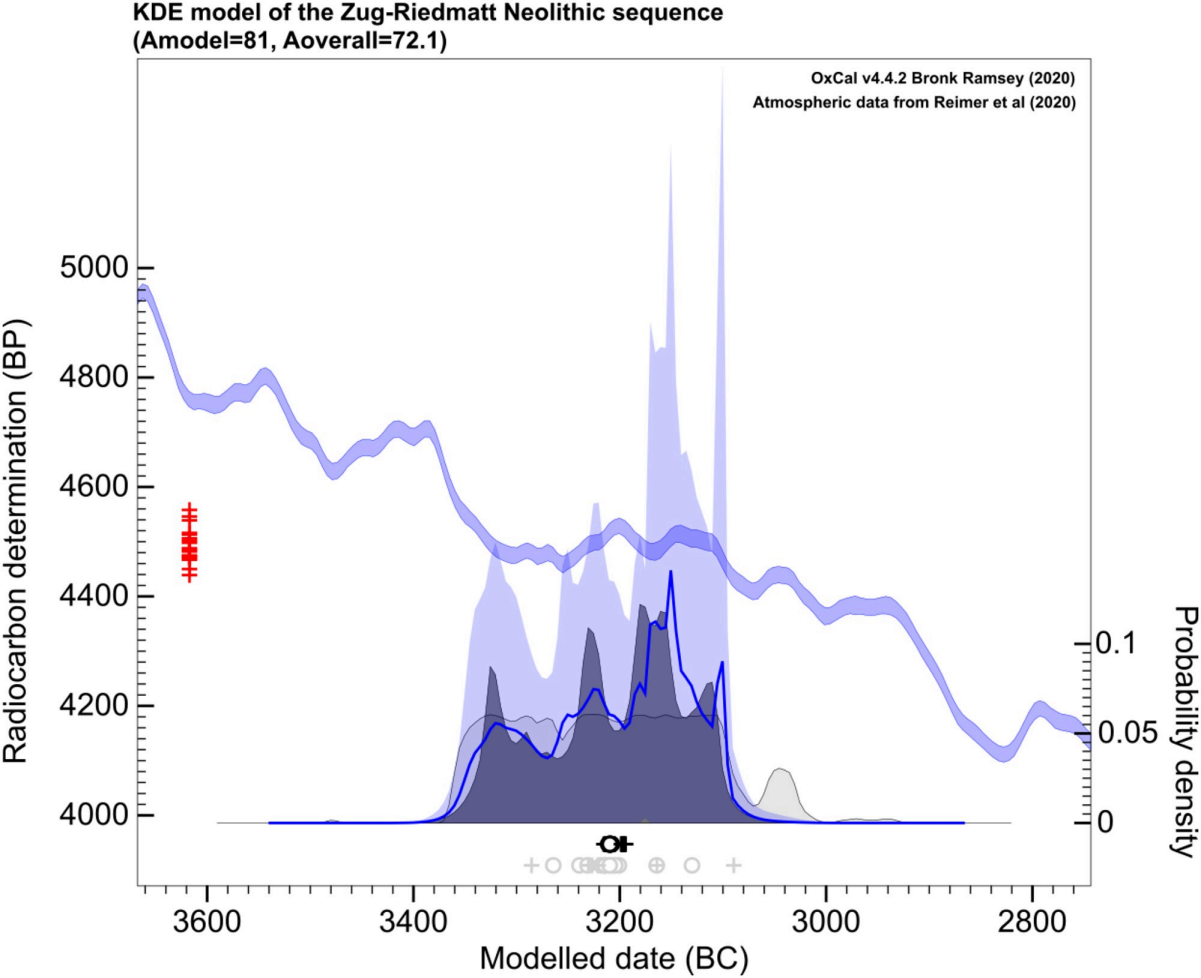
KDE model of the chronological sequence of Zug-Riedmatt.

The results of the KDE model (Amodel = 81, Aoverall = 72.1) indicate that the occupations of Zug-Riedmatt ranged from 3281 to 3149 cal BC (95.4%) (Fig 17). They also allowed to advance a Summed Calibrated Radiocarbon Date Probability Distribution (SCDPD) model as the mean ± 1σ for the snapshots of the KDE distribution generated during the Markov chain Monte Carlo (MCMC) process yielded clear indications that the most recent tail of the SCDPD must be interpreted as background noise (see Bronk Ramsey 2017: 1821 for further details on interpreting the graph). The probability of the distribution of the KDE qualifies the uniformity of the SCPDP, generating different degrees with the highest density of probability concentrated at ca. 3200–3100 cal BC (95.4%) (S2 Table).

**Fig 18:**
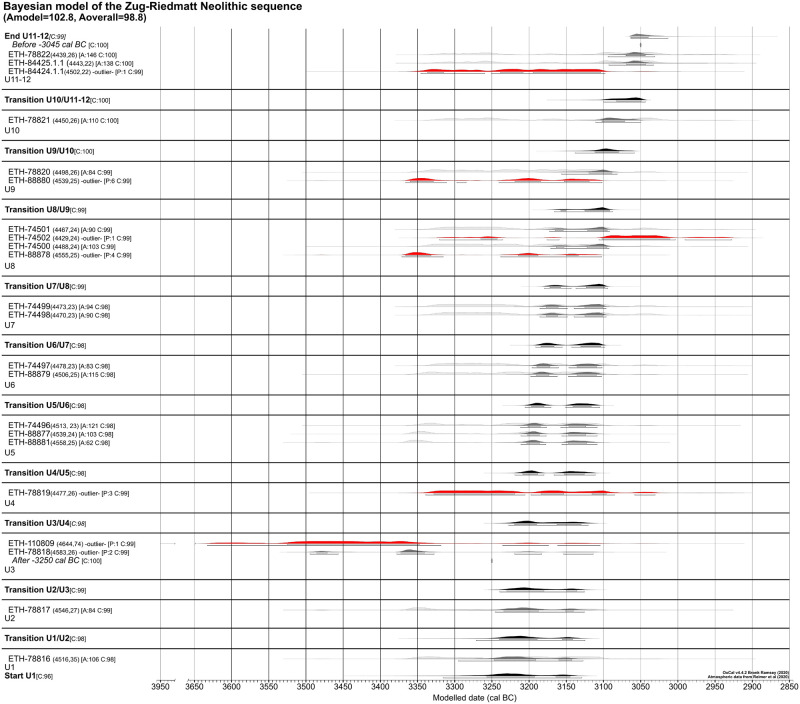
Bayesian model of the datings of the stratigraphical units of Zug-Riedmatt.

The results of the Bayesian modelling are statistically valid (Amodel = 102.8, Aoverall = 98.8) (Fig 18) as it illustrates a broad chronology stretching from ca. 3220 to ca. 3045 cal BC (95.4%). The episodes of the individual stratigraphical units are as follows: unit 3 ca. 3198–3185 cal BC (95.4%), unit 5 ca. 3160–3144 cal BC (95.4%), unit 6 ca. 3144–3133 cal BC (95.4%) and unit 8 ca. 3121–3111 cal BC (95.4%). Among the available datings for the model there are nonetheless outliers yielding radiocarbon values marked by age reversals that do not fit into with stratigraphic and chronological sequence ([70]) (S2 Table).

### 3.6 Seasonal climate and crop growing conditions based on cereal isotope values

**Fig 19:**
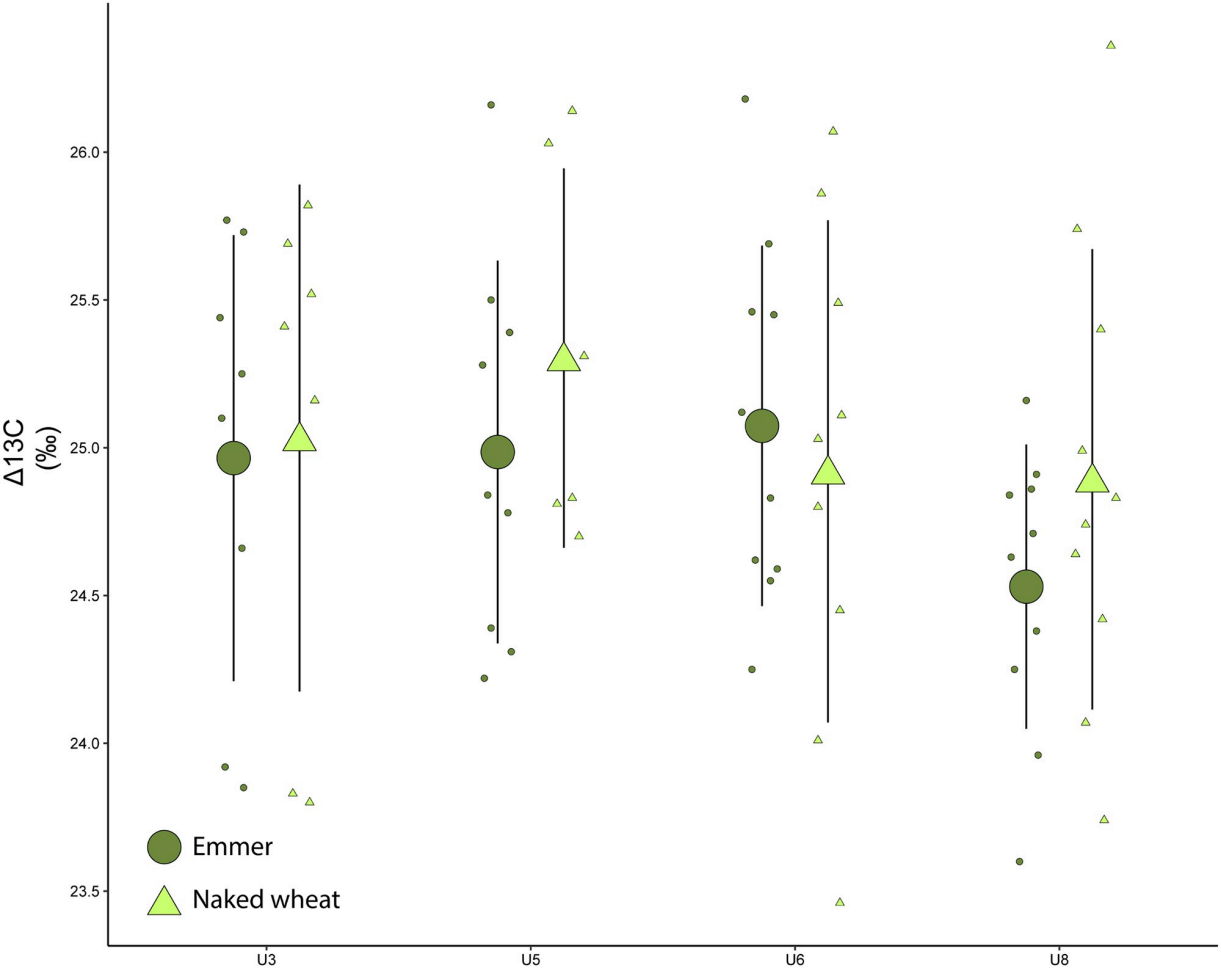
Δ^13^C values of emmer and naked wheat from units 3, 5, 6 and 8 of Zug-Riedmatt according to crop types. The higher Δ^13^C values suggest greater water availability.

Fig 19 indicates that the results of the Δ^13^C analyses of emmer and naked wheat at the species level overtime are practically homogeneous in spite of certain stages of variability that also differ at the species level. The results of emmer wheat (R = -0.82, p-value = 0.18) of U3, U5 and U6 coincide more or less, while those of U8 are lower. The analyses of naked wheat (R = -0.52, p-value = 0.48) of U3, U6 and U8, in turn, are likewise similar while those of U5 are higher (S3 Table).

From a diachronic perspective, the results of emmer and naked wheat of units 3 (av = 24.97, sd = 0.71; av = 25.03, sd = 0.79), 5 (av = 24.99, sd = 0.61; av = 25.30, sd = 0.59), 6 (av = 25.07, sd = 0.58; av = 25.08, sd = 0.61) and 8 (av = 24.53, sd = 0.46; av = 24.61, sd = 1.02), subsequent to a one-way ANOVA test, reveal no significant differences (p-value = 0.08). On the other hand, the comparison of units 5 and 8, and 6 and 8, yield p-values that are lower than 0.05. From the synchronous perspective, all tests of significance between species by units yielded p-values above 0.05.

## 4. Discussion

### 4.1 Methodology

#### 4.1.1 Research limitations

The number of analysed samples collected by means of monolith columns of the different units varied greatly according to the thickness of the settlement layers. Unit 8 yielded the highest number due to its >40 samples from eight monolith columns. This value is considered the minimum to obtain representative results when the sample volume is small ([25], 331). Unit 6 with its >30 samples from seven monolith columns is still well-represented. Units 3 and 5, in turn, with less than 20 samples (respectively six and five monolith columns) were not as well-characterised as the previous two. By contrast, only two samples per unit were analysed among those collected though bulk sampling among all units except unit 5. While a large number of specimens was identified among every sample and fraction, the units might likewise not be as ideally characterised by bulk samples. Approximately eight samples collected by bulk sampling are required to yield a good representation of the most common cultivars and gathered plants inside a structure ([24]).

#### 4.1.2 Comparison of small-volume monolith and large-volume bulk samples

The mean density and number of remains were usually greater among the bulk samples. This is in line with the findings of similar research carried out for the settlement of Arbon Bleiche 3 ([32], 413–414; [33], 84–85). Furthermore, the study of layer 13 of the roughly contemporary site of Parkhaus Opéra (Lake Zürich) revealed no significant differences among the mean values obtained from either large and small (bulk) samples, whereas there were significant differences at the level of the sample ([25]). The monolith samples of Zug-Riedmatt, in turn, revealed a greater diversity, while the evenness (Shannon Index of Evenness) among the bulk samples was higher in three units. The index of evenness of the bulk samples therefore suggests the assemblages to be slightly more regularly distributed than they actually are. This indicates that the rare plant diaspores only present in small amounts might be underrepresented by this sampling technique. However, the result could also stem either from a lesser number of samples and of sampling positions for this type or that those samples collected by the bulk technique were mixed with materials from underlying and overlying layers.

The fact of a higher mean density among the bulk samples mainly derives from the more common taxa that appear in great numbers in all samples (mostly cultivars and woodland plants, as illustrated in Fig 9 and 13X). Concerning these common taxa, the order of the units (highest to lowest density) of the cultivars among the monolith samplings was the same or similar to those collected by the bulk technique (except flax). The order of gathered fruit and other common taxa such as silver fir (*Abies alba*), greater plantain (*Plantago major*), mouse-ear (*Cerastium sp*.), vervain (*Verbena officinalis*) and thyme-leaved sandwort (*Arenaria sepyllifolia*), in turn, differed more often between the two sampling types. The differences of composition between the bulk and monolith samples therefore could indicate that cultivars were frequently dispersed throughout the settlement while others might have been concentrated only in specific sectors (e.g., where they were processed or grown).

The differences between the two sampling techniques are less pronounced when observing taxa proportion rather than density. Most of the values of cultivated taxa and common gathered fruit reveal variations between the monolith and bulk sampling techniques that align with the standard error range (except for barley in unit 8). The differences between the two sampling types concerning calories is also rarely greater than between the units (except for the gathered plants of units 5 and 6). In line with these results are the CA findings (of cultivars and gathered fruits) which demonstrate that bulk samples are more or less grouped with their monolith counterparts of the same units. The characterisation of both cultivated plants and gathered fruits are similar among both monolith and bulk samples, with bulk samples containing higher densities while those of the monolith type reveal a greater taxa diversity.

Despite being based on modest numbers, the order of density of the charred finds (from highest to lowest) remained consistent throughout the monolith and bulk samples. In the case of charred cereals (both grains and chaff), this could stem from taphonomic factors. Different sedimentation or post-sedimentation dynamics could have influenced the deposition of charred cereals throughout the site. In the case of charred gathered fruits, they attained their highest density in both the monolith and bulk samples of unit 5. This is the unit containing the ‘bone midden’, and middens in general are known to contain greater amounts of charred plants (e.g., [71–74]). Why this is not the case for the charred cereals, and whether these results are representative when considering the site’s low number of charred finds, remains unclear.

#### 4.1.3 Which plant remains are affected the most by the differences between the two sample types?

Crop plants reveal no pronounced dissimilarities between the two sample types. The findings nonetheless indicate differences in the order (highest to lowest density) among flax and einkorn wheat. Imbalances among gathered fruits are more apparent and the order of density differed in most cases.

Certain ecological groups containing taxa that are rare appear in much greater density among all four of the units sampled by the monolith technique. These include reed bed and sedge swamp plants, unassigned wetland plants, grassland plants, winter crop annual and flax weeds, perennial ruderals and potentially cultivated plants. Groups containing the most common taxa of the site (woodland plants, cereals, pulses and oil and fibre plants), on the other hand, appear in much higher densities in all of the four tested units sampled by the bulk technique. These results could be influenced by the differing numbers of samples analysed per technique, as rare plants might be less well-represented among bulk samples due to their lower number and the very limited number of sampling positions.

According to the findings of this study, the site’s most common plant remains can be identified by both types of sampling. However, their density is underestimated among the monolith samples even when analysed in great number. These results align with the comparisons of the two types of sampling carried out at Arbon Bleiche 3 ([32], 413–414; [33], 84–85). However, this result does not normally affect proportions of cultivated plants and gathered fruits, and this difference should therefore not pose a problem when investigating economic questions. However, concerning plants occurring in clusters or that are rare, it is essential to collect enough samples throughout the whole area of a site (regardless of the sample type), even when the excavation is modest such as that of Zug-Riedmatt. When comparing the results of the bulk and monolith samples of unit 8 (marked by a good characterisation through monolith samples), it is possible to advance that two bulk samples could suffice to characterise the most common cultivated taxa. Yet it is clear that all the other taxa (among them charred remains of common cultivated species) will be less well-represented when collecting fewer samples and when it is not possible to differentiate between structures within a site—compare to the eight bulk samples per structure recommended by [24]. It is possible that the number of samples required for a good representation of the most common cultivated taxa varies according to site or stratigraphic layer. Until this question is resolved, it is better to sample more rather than less, especially when the research questions go beyond economic issues. As to the samples of Zug-Riedmatt, this study has concluded that the dataset is of high quality and representative of the changes, particularly those regarding cultivated plants, that took place throughout the site’s occupation.

### 4.2 Chronology

The results of the latest radiocarbon datings place the different layers of occupation of Zug-Riedmatt into a Neolithic sequence ranging between ca. 3200 and 3100 cal BC. Each of the phases of human occupation were highly concentrated, represented by sequences of approximately 10 years separated by brief interruptions of activity, never more than a decade. Similar temporal dynamics have been observed at other wetland settlements (e.g., [75, 76]), possibly linked to woodland over-exploitation ([77]) or other events such as short-term periods of flooding, and specialised or seasonal activities. These short lived settlements interspersed by periods of inactivity must be contextualised within the reality of the different occupations towards the end of the 4^th^ millennium cal BC of the northernmost shore of Lake Zug marked by abundant wetland settlements (see Fig 2), many presumably ‘synchronous’ ([37], 261). The archaeobotanical findings per unit of the current study of Zug-Riedmatt therefore correspond at best to economic practices ranging on an average to about 10 years. They thus offer four ‘snapshots’ of a Late Neolithic occupation extending over the course of about five decades between 3200 and 3100 cal BC.

### 4.3 The Late Neolithic plant economy of Zug-Riedmatt

#### 4.3.1 Research limitations

Due to being embedded in two specific research projects, the current study has necessarily adopted a focus restricted to archaeobotanical questions. The samples gathered at Zug-Riedmatt have also been the object of analysis of other disciplines (archaeology, palynology, micromorphology, ichthyology, osteology, entomology, parasitology, palynofacies, and geochemistry). The disparate rates of progress of these different disciplines, the absence of an integrated transdisciplinary discussion, and the lack of an adequate assessment of the interrelations precludes a transdisciplinary approach to the research questions. There are nonetheless preliminary indications that the clear differences reported here between units 3–5 and units 6–8 are also observed by other disciplines. Only when the interconnections and interrelationships of data from the various disciplines are better understood will it be possible to fully decipher the current findings, which could stem from human intervention and/or broader climatic/ecological changes affecting the environment of the site (especially its surrounding delta). Advancing relevant findings thus requires expanding the understanding of the microregion to include factors such as soil suitability, landscape demographic impact (e.g., land clearing attested through palynology and dendrochronology) and their association with demographic fluctuations. These types of considerations must assume perspectives that clearly go beyond individual sites. Only then will it be possible to develop different explanatory models to gain a better grasp of the impact of a variety of factors (differences in subsistence, land use, climate and climatic fluctuations and seasonal focal points) and their interrelationships. These issues thus illustrate the urgent need for further research.

#### 4.3.2 Crop plants and gathered fruits over time

Opium poppy, flax and emmer wheat were Zug-Riedmatt’s most common crop plants. Waterlogged cereal grains (preserved in the form of testa fragments) were also numerous. These plants reveal high densities and proportions in every unit and layer, suggesting they were in use throughout every phase. However, their frequencies fluctuated over time, revealing greater similarities with the earlier units 3 and 5 dominated by oil plants, cereal testa remains and emmer wheat chaff, as opposed to those of the later units 6 and especially 8 dominated by naked wheat chaff and peas. Charred cereal grains and cereal chaff of all species were likewise more frequent in the later units 6 and 8.

Two potentially cultivated plants, celery and dill, also only appeared in units 6 and 8 albeit in very low densities and only among the monolith samples. Moreover, it is not certain that einkorn wheat, which does not follow this trend and is rare throughout all units, was actually cultivated for consumption. It was potentially grown as a sort of last resort after the other options failed or as an admixture with other cultivated cereals (a general phenomenon recorded elsewhere in the Late Neolithic such as [39]).

The most common gathered fruits were raspberries, wild strawberries, crab apples, blackberries, hazelnuts, acorns, winter cherries, rose hips and beechnuts. It is hard to distinguish general trends between the units as the values differ not only between species and types of remains but between the monolith and bulk samples. In general, no great differences are observed between units as to the values of density and proportion of the most common gathered fruits. Less common gathered fruits, in turn, at time reveal peaks in one unit. This is the case of acorns in the monolith samples of unit 8, beechnuts in the monolith samples of unit 6 (although strongly influenced by as single sample with a very high density) and winter cherries among the bulk samples of unit 5. Concerning calories, the composition of taxa per unit did not differ much except for the monolith samples of unit 6 due to the concentration of beechnuts cited above. In all other cases, hazelnut offered the greatest amount of caloric input.

**Fig 20:**
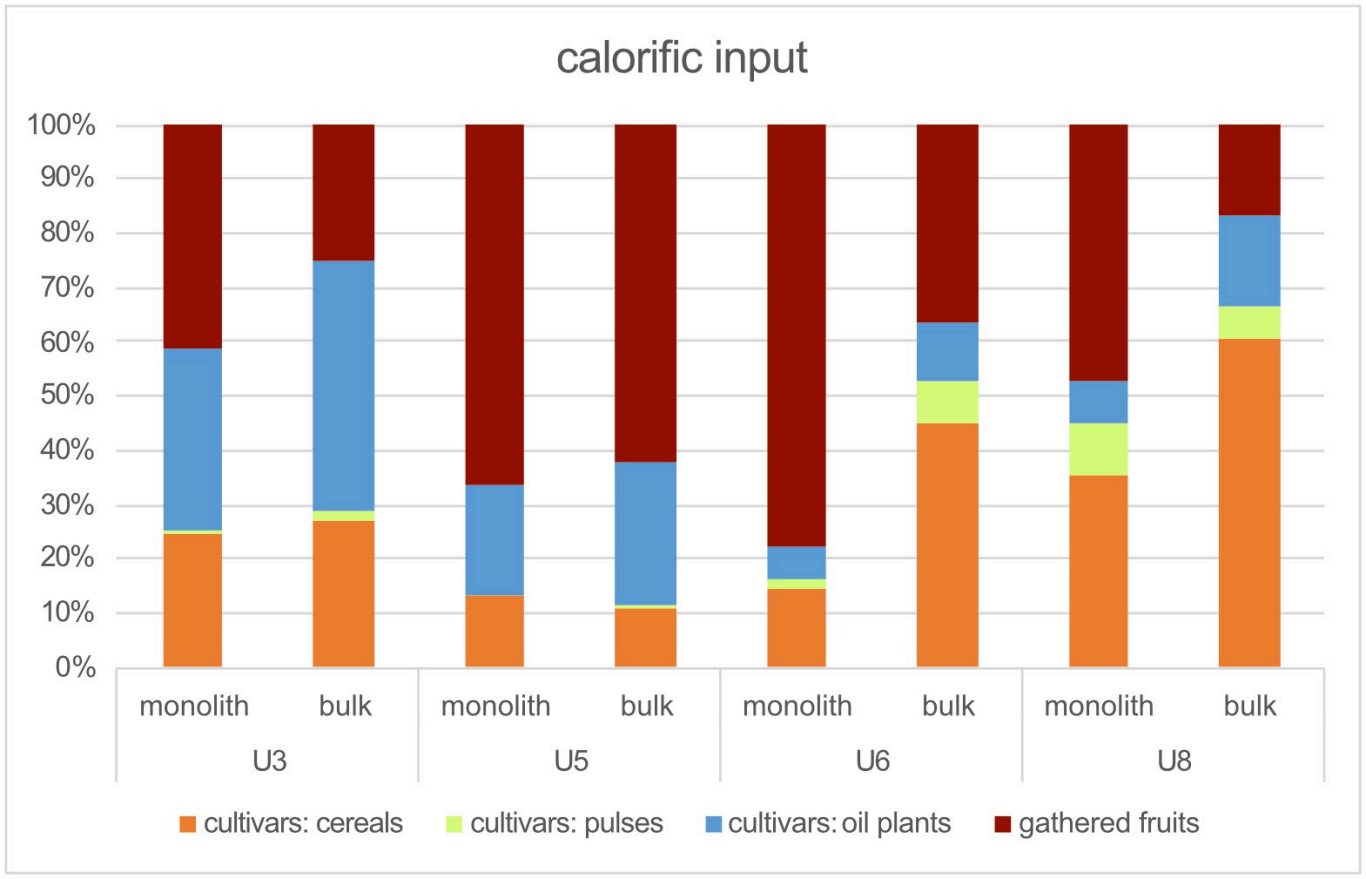
Comparison of the caloric input of cultivars and gathered fruit.

Certain differences between units arise when comparing the caloric input between cultivars and gathered fruits (Fig 20). The caloric input stemming from gathered fruits has its highest values in unit 5 (~64%)—coinciding with the great concentration of wild animal bones—as well as in the monolith samples of unit 6 (78%, although this stems solely from the beechnut concentration cited above). Unit 5 and its high proportion of wild elements could either correspond to a special event (a sort of a feast or a seasonal hunting event) or to a period of crisis when Zug-Riedmatt’s inhabitants may have had to rely largely on wild resources for other reasons such as poor harvests (see e.g., [78]). Furthermore, the fact that unit 6 still partly follows this pattern suggests that this potential crisis was not necessarily an isolated event.

The contribution to the diet of gathered plants in units 3, 8 and among the bulk samples of unit 6 is less than 50%. It is therefore possible to arrive at the general assumption that fruits represented by organic deposits were gathered in large amounts throughout all settlement phases and that the amounts of the different taxa also hardly changed. It is possible that there were local differences within settlements as is recorded at other wetland sites such as Arbon Bleiche 3 ([27]) and layer 13 of Zürich-Parkhaus Opéra ([74]), However, these could not be identified at Zug-Riedmatt due to the limited excavated area and the lack of information as to its buildings.

#### 4.3.3 Crop management over time

**Fig 21:**
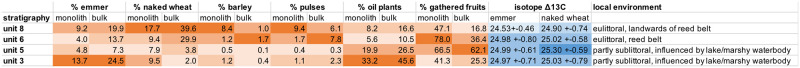
Summary of the different measured indicators of units 3, 5, 6 and 8 of Zug-Riedmatt. Percentages of caloric input, isotope values and local environmental conditions (based on [8]).

The Δ^13^C values of emmer and naked wheat of units 3, 5, 6 and 8 do not reveal significant differences (Fig 21), suggesting that they grew in the same fields or under similar conditions. The variability within units can be explained by in-field distinctions or changes in inter-annual precipitation (since the study involves ca. 10-year slices). What is also relevant to the analysis is that the highest values of naked wheat in unit 5 suggest greater water availability during the ear formation and the lowest values for emmer wheat in unit 8 slightly less water availability during the ear formation. Isotopes alone cannot define whether local conditions changed or whether this reflects less spring precipitation in unit 8. This will be discussed in the light of other evidence.

#### 4.3.4 Possible causes for the changes of plant economy at Zug-Riedmatt

The analyses highlight that a major change took place at Zug-Riedmat marked by a shift from a diet mainly founded on opium poppy, flax, emmer wheat combined with hazelnut (units 3 and 5) to one predominantly based on cereals, notably naked and emmer wheat, barley, as well as pea (units 6 and 8) combined with hazelnut, beechnut (unit 6) and acorn (unit 8) (Fig 21). To what extent do these fluctuations reflect climate or local environmental changes? The change of local conditions observed among the aquatic and wetland plants suggests that the settlement might have been situated in a sublittoral environment or that it was subjected to frequent flooding (short-term or seasonal events) during the formation of unit 3, whereas during the formation of units 6 and 8 it found itself in the eulittoral zone (unit 6 potentially inside and unit 8 landwards of the reed belt) or in calm waterbodies not directly linked to the lake ([8], Ismail-Meyer et al., in prep.).

**Fig 22:**
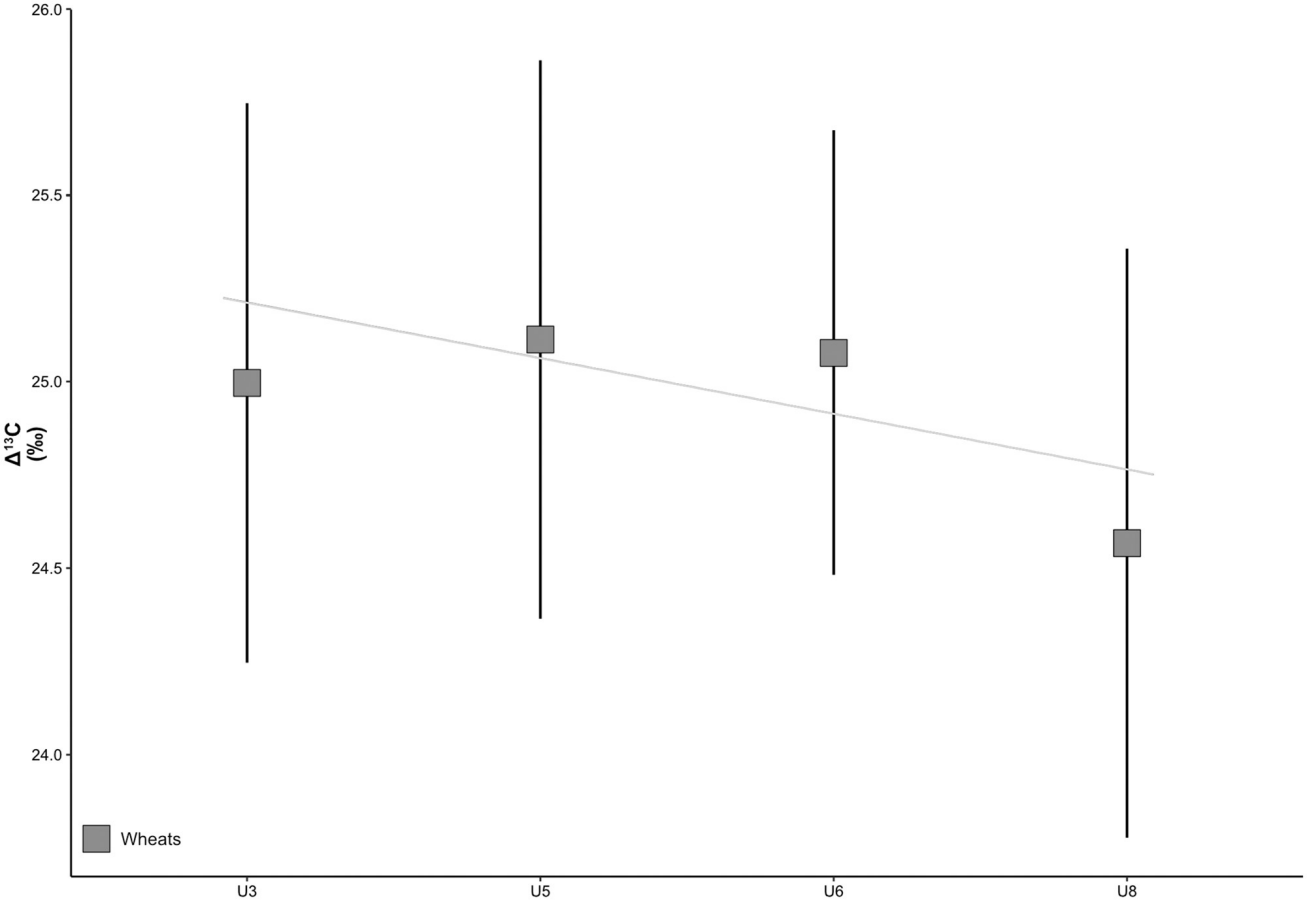
Δ^13^C values of wheats from units 3, 5, 6 and 8 of Zug-Riedmatt and the regression line. The higher Δ^13^C values suggest greater water availability.

A progressive local environmental change towards a less humid environment based on the study of aquatic plants and wetlands is also confirmed by Δ^13^C analyses of cereals (Fig 22). The earlier units 3 and especially 5, corresponding to a period when the site was more influenced by the lake, reveal the wettest conditions for cereal cultivation. On the other hand, when the site’s environment changed from sublittoral to eulittoral, at least in unit 8, cereal growing conditions became drier. However, the available Δ^13^C values are always very high. When assuming that the agricultural fields were close to the settlements themselves, this could reflect very local changes. When assuming that the agricultural fields were distant from the site, which was situated in the highly dynamic (thus unsuitable for growing crops) delta of the Lorze River along Lake Zug ([37]), the changes could relate to more widespread climatic factors taking place beyond the immediate lakeshore and delta. The increase of naked wheat also falls in line with drier conditions. In either case, this suggests that the economy of the site had to be flexible and adaptable in order to remain resilient. This was probably achieved by means of having access to a broad spectrum of crops and abundant nearby wild resources (wild plants, game and fish/amphibians).

The factors affecting the data advanced above include the limitation stemming from the modest excavated area and the nature of each unit. The dataset appears nonetheless to be robust enough to consider it as representative of the issues brought up by this study, which has not attempted to advance an exact quantification of each gathered plant, but reflect on them in a global manner.

To date there is no evidence of the presence of pest insects at Zug-Riedmatt in spite of the fact that the pea weevil (*Bruchus pisorum*) was identified between 3176–3153 BC in layer 13 of the nearby site of Zürich-Parkhaus Opéra ([79]). It is thus unlikely that the change in the cereal spectrum was due to a pest outbreak. Moreover, cereal pests appear to vanish from central Europe by the mid-5^th^ millennium cal BC ([80]). There is also no trend towards the cultivation of naked cereals in contemporary nearby sites.

Taphonomic factors may have also influenced to a certain degree the layers of Zug-Riedmatt. The study of layer 13 of Zürich-Parkhaus Opéra, situated in sublittoral conditions, advanced that barley and naked wheat chaff, as well as flax capsules, were concentrated in the site’s landwards sector. They could possibly have been subsequently transported and deposited by the drift line. The same was not observed for emmer wheat chaff, whose spread might be linked to different processing methods (domestic dehusking ([81]) contrary to naked cereals) and thus subjected to a different taphonomic process ([74]). These factors, however, cannot explain all the changes observed at the site.

The finds of unit 5 led the authors of [37] to hypothesise that Zug-Riedmatt may have experienced different seasonal ventures. The archaeozoological finds of this unit presumably reveal evidence of seasonal events. The first is a focus on activities associated with spring/early summer (frog, small fish, red deer drive hunt) followed by pursuits linked to winter (winter fishing; [13]). The plant spectrum of this unit is also particular as it comprises great numbers of gathered fruits and evidence of the Cretan catchfly (*Silene cretica*, [38]), finds supporting the notion of a special event or events. This unit deserves a more detailed transdisciplinary examination to evaluate its unique characteristics. However, organic units 3, 6 and 8 contained crop plants and gathered fruits with similar harvesting times. Unless finds of short specialised events were not preserved in enough detail for their recognition, it is doubtful that seasonality can fully explain the changes observed between these units. Moreover, a transdisciplinary study of a “reference” monolith section of Zug-Riedmatt has also not been able to clearly distinguish seasonal patterns (Ismail-Meyer et al. in prep.).

The evidence does not support the view that the changes in the crop spectrum necessarily reflect crop choice, although the oil plants have additional functions besides nutrition (flax for textile fibres, opium poppy for medicine or for mind-altering). The study indicates that all crops are present in all phases and that this pattern might be best explained by changes in either the local environmental or more widespread climatic conditions as well as the dynamics of the settling process. The initial phases of Zug-Riedmatt reveal greater links to hunting, a notion bolstered by finds from other lakeshore settlements (e.g., [82]), which served to compensate the lack of domestic animal protein. Evidence of parasites suggests a key role of fishing linked to unit 3 ([83]), while unit 5 mostly reflects resorting intensively to wild resources (gathered plants, game and fish, see also [12]). It is possible to speculate that all crop plants were grown each year up to a certain scale, while gathered fruits were collected with the same intensity, and the residents simply adapted to the success of each yield. This could have depended, for example, on manure availability, flooding and other extreme meteorological events. This suggests that if more cereals were available, then more were consumed. And on the contrary, if not, oil plants or gathered fruits might have played a more important role. This is a resilient strategy to deal with poor harvests of certain crops. Yet in order to have access to these alternatives, the landscape around the settlement could have been manipulated to improve wild plant gathering (niche construction, see e.g., [6, 84]), actions that probably also took place in Neolithic communities [2, 3]). The greater number of grassland plants identified at Zug-Riedmatt in units 6 and 8 ([38]) could point to an opening of the landscape potentially linked to this type of management.

Pea values increased starting in unit 6. Pea could have served in rotation or in combination with other crop plants to contribute nutrients to the soil. Meanwhile, the drier local environmental conditions observed for units 6 and 8 cited above (Fig 21) could have enabled the growth of more demanding crop species such as naked wheat if the fields were near the settlement. The parasitological analyses also suggest rise of cattle breeding in units 6 and 8 [83] (although pig retained its overall dominance [85]). Cattle thus appear to spend more time in the settlement’s immediate surroundings (wet grassland, which increased significantly in unit 8 and served at the time as one of the few naturally occurring habitats offering grazing). This could likewise suggest that more manure was available in the periods represented by these units for more demanding crops.

These resilience strategies of Zug-Riedmatt could thus lead to differing priorities associated with the crop plants within the periods represented by the different units and higher proportions of gathered fruits compared to crop plants during periods marked by unfavourable growing conditions. Meanwhile, the evidence of the total composition of gathered fruits reveals no drastic changes over time.

## 5. Conclusions

### Methodology findings

Plant macrofossils appearing regularly at an archaeological site can be identified in both monolith and bulk samples. Their density, however, will be underestimated in monolith samples even when the number of analysed samples is great. This does not normally affect proportions of cultivated plants and gathered fruits and should therefore not pose a problem when investigating economic issues. Care must be taken when collecting bulk samples in multiphase sites in order to avoid mixing material from the over- or underlying layers. Thus to render possible the identification of the remains of plants that occur in clusters or plant types that are rare, it is likewise essential to collect enough samples throughout all the site and from all the features regardless of the type of sampling (see for example recommendations in [33]).

### Economic strategies

The findings of this archaeobotanical and isotope examination of organic samples collected from the different phases of occupation of the Late Neolithic settlement of Zug-Riedmatt suggest that major changes took place among crop plants. The lower units of the stratigraphy reveal a dominance of oil plants and emmer wheat. Naked cereals (wheat and barley) and pea subsequently gain in importance over the course of a few decades. The evidence also points to a change in the role of gathered fruits as to caloric input while their total composition remained more or less the same over time. The findings lead to the hypothesis that these differences mostly reflect a resilience strategy allowing the inhabitants to react to short term fluctuations of differing nature (especially the availability of water in the last settlement phase). While the landscapes of today are for the most part marked by permanent settlements, ameliorated fields, channelled waters and wetland drainage, the Neolithic lakeshore settlements found themselves in highly dynamic, ever-changing environments. The evidence gleaned from this study suggests that Zug-Riedmatt’s inhabitants responded to these fluctuations by adopting a resilient agricultural strategy of growing different crop plants while simultaneously gathering wild plants in nearby marshes and woodlands in addition to hunting and fishing, hence adapting their diet to the yield of each harvest depending on local environmental conditions.

## Supporting information

S1 Table Table of the values serving to calculate calorie input. (CSV)

S2 Table Table of the AMS radiocarbon datings of waterlogged finds from Zug-Riedmatt (nd = undetermined) (complete list). (CSV)

S3 Table Table of the Δ13C values of uncharred chaff samples from Zug-Riedmatt, units 3, 5, 6 and 8 dated to the Late Neolithic (Horgen Culture) (complete list). (CSV)

S4 Table Table listing the density values of all samples and the codes of the taxa serving for the correspondence analysis. (CSV)
